# Benzimidazole scaffold as a potent anticancer agent with different mechanisms of action (2016–2023)

**DOI:** 10.1007/s11030-024-10907-8

**Published:** 2024-07-20

**Authors:** Fatma Fouad Hagar, Samar H. Abbas, Eman Atef, Dalia Abdelhamid, Mohamed Abdel‐Aziz

**Affiliations:** 1 Medicinal Chemistry Department, Faculty of Pharmacy, Minia University, Minia, Egypt; 2 College of Pharmacy, West Coast University, Los Angeles, CA USA; 3 Raabe College of Pharmacy, Ohio Northern University, Ohio, USA

**Keywords:** Benzimidazole, Anticancer, Hybrid, Metal complex, Mechanism of action

## Abstract

Benzimidazole scaffolds have potent anticancer activity due to their structure similarity to nucleoside. In addition, benzimidazoles could function as hydrogen donors or acceptors and bind to different drug targets that participate in cancer progression. The literature had many anticancer agents containing benzimidazole cores that gained much interest. Provoked by our endless interest in benzimidazoles as anticancer agents, we summarized the successful trials of the benzimidazole scaffolds in this concern. Moreover, we discuss the substantial opportunities in cancer treatment using benzimidazole-based drugs that may direct medicinal chemists for a compelling future design of more active chemotherapeutic agents with potential clinical applications. The uniqueness of this work lies in the highlighted benzimidazole scaffold hybridization with different molecules and benzimidazole-metal complexes, detailed mechanisms of action, and the IC_50_ of the developed compounds determined by different laboratories after 2015.

## Introduction

The number of cancer survivors is on the rise, along with the applied screening protocols that have helped in the early detection and advancement of treatment. The latter is due to the ceaseless evaluation of newly developed molecules and rigorous persistent investigations to improve the performance of approved anticancer drugs. These are the ways to advance cancer treatment drug discovery.

**Fig. 1:**
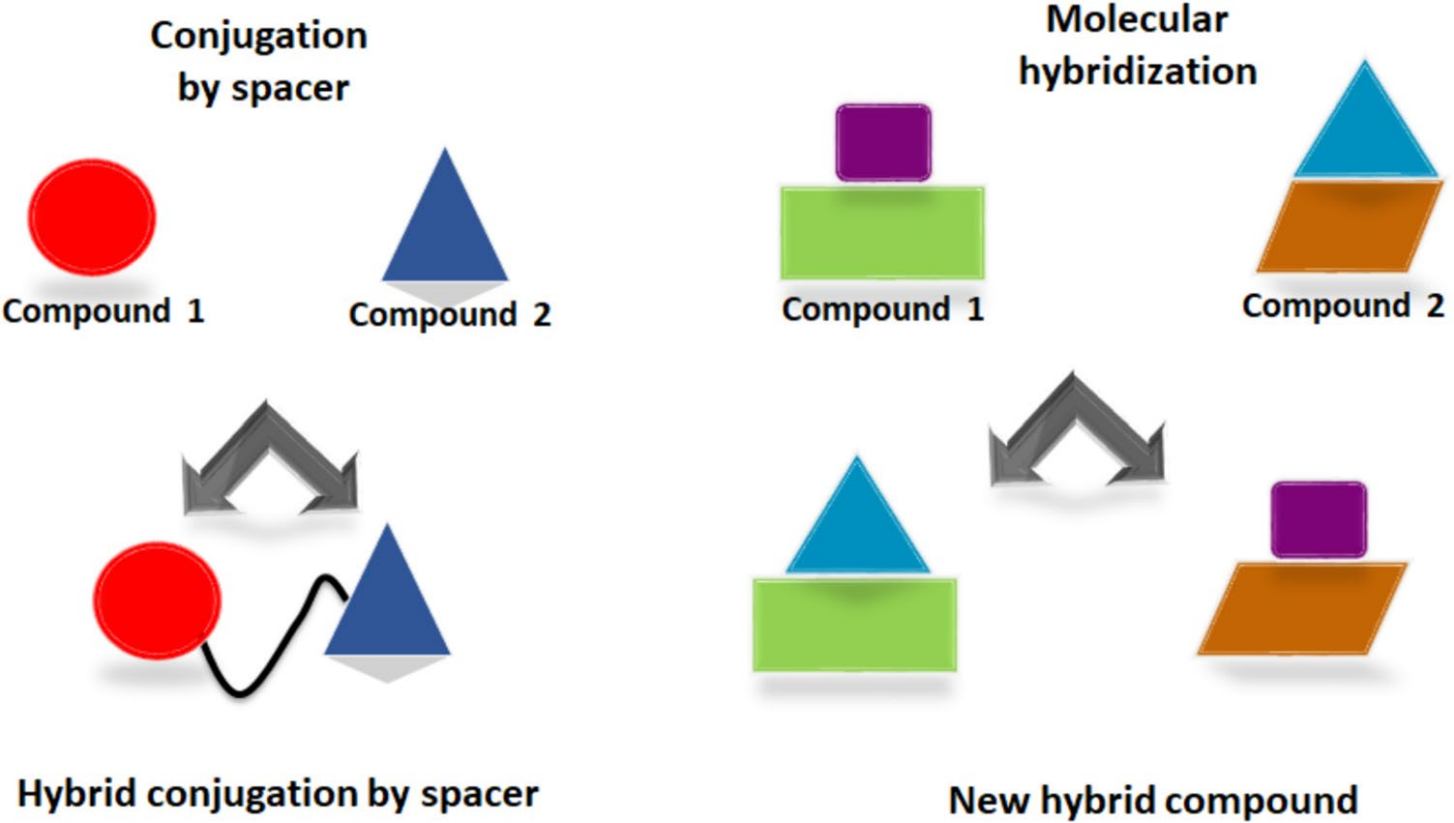
An illustration of the molecular hybridization and design of the new scaffold

Cancer is a significant public health concern and a top cause of death worldwide. It is defined as abnormal cell growth leading to new neoplasms that spread around different body parts [1]. Cancer is very similar to parasitic organisms because it affects normal cells, the immune system, and blood vessels [2, 3]. However, the projected total number of new cancer cases worldwide is 21.7 million, and up to 13 million deaths are predicted to occur by 2030 [4]. There are many leading factors associated with cancer, including but not limited to inherited mutations, hormones, exposure to radiation, environmental contaminants, and lifestyle factors, such as obesity, smoking, physical inactivity, and alcohol consumption [5]. One strategy for identifying new anticancer agents involves the use of metal complexation or molecular hybridization techniques, which involve the combination of two or more pharmacophoric moieties to form new hybrids with improved physiochemical and pharmacokinetic properties compared with those of the parent pharmacophores [6, 7]. Hybridization between two pharmacophores can be performed either by linking subunits directly [8] or by connecting them through a spacer moiety utilizing alkylation reactions [9, 10] (Fig. 1).

**Fig. 2:**
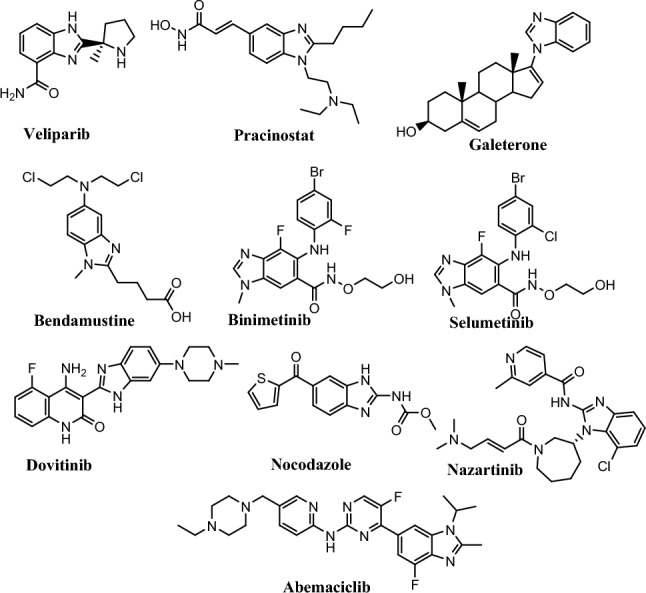
Examples of marketed anticancer drugs containing benzimidazole rings

The benzimidazole nucleus is a valuable class of organic heterocyclic compounds. The reduction of 2-nitro-4-methylacetanilide in 1872 led to benzimidazole's discovery by forming 2, 5- or 2,6-dimethyl benzimidazoles [11]. Moreover, an integral part of the structure of vitamin B12 is 5,6-dimethyl-1-(α-D-ribofuranosyl) benzimidazole [12]. Furthermore, benzimidazoles have potent DNA-binding activity because their structure is similar to natural purines. In addition, the benzimidazole nucleus is a building block of many marketed anticancer agents, such as bendamustine (Treanda) [13], nocodazole [14], dovitinib [15], binimetinib [16], selumetinib [17], abemaciclib [18], veliparib [19], pracinostat [20], galeterone [21], and nazartinib [22] (Fig. 2).

**Fig. 3:**
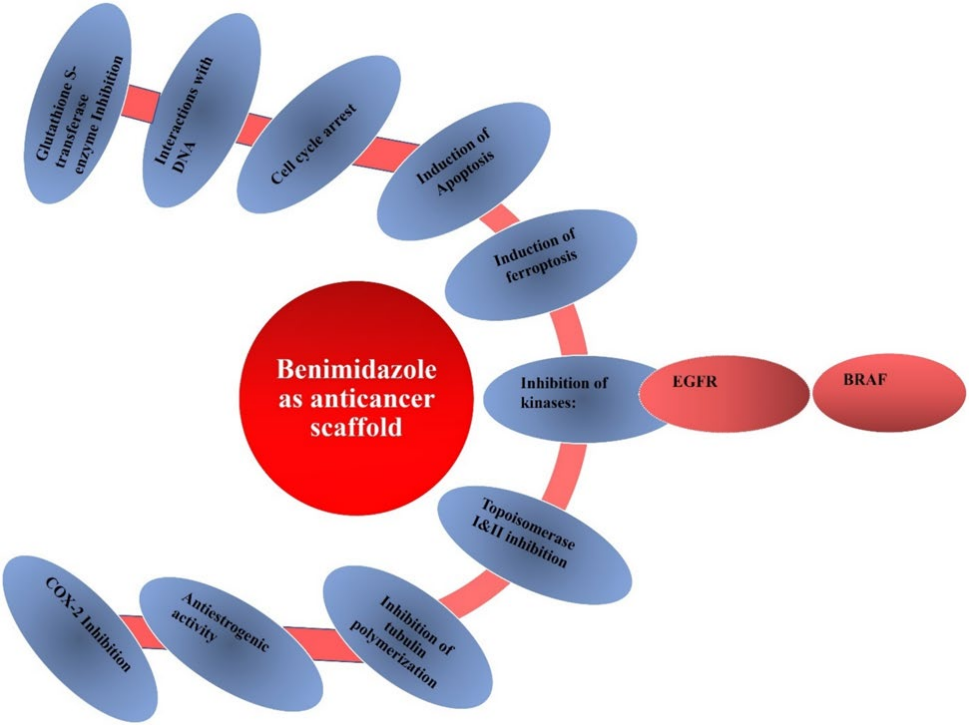
Illustration chart of different mechanisms of benzimidazole scaffolds as anticancer agents

Many benzimidazole derivatives in the literature have also displayed promising anticancer activity against different cancer cells. These activities may be attributed to various mechanisms (Fig. 3) such as the induction of apoptosis [23, 24] or ferroptosis [25]; blockade of cell cycle progression at different stages [26, 27]; DNA interactions [28, 29]; and inhibition of one or more of the following targets: epidermal growth factor receptor (EGFR) [30], B-Raf proto-oncogene, serine/threonine kinase (BRAF) [31], tubulin polymerization [32], and topoisomerase (TOPO) I & II [33, 34].

Benzimidazole nucleus can be synthesized through a condensation reaction of 1,2-benzenediamines with aldehydes without a catalyst or using nanomaterial, acid, metal, sodium metabisulfite, or natural sources catalysis. Also, it can be prepared from condensation of 1,2-benzenediamine or its analogs with primary alcohols, primary amines, carboxylic acids, or β-keto esters. Moreover, it can be obtained by reacting *o*-nitroanilines with aldehydes using different catalysis conditions [35].

**Fig. 4:**
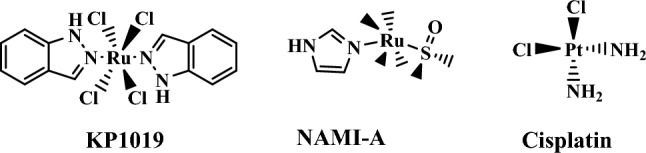
Examples of anticancer agents containing metal complexes

By hybridizing the benzimidazole nucleus with different molecules, scientists could generate synergetic effects, reduce side effects, and minimize drug resistance. In addition, metal complexes are metal-based compounds with unique structures formed from metal core coordinates with different ligands. Some metals are essential for the human body to perform critical biochemical processes [36]. Zinc, iron, manganese, and copper are imperative examples of these metals of which deficiency can lead to the manifestation of some diseases, such as anemia, heart problems, and growth retardation [37]. Researchers have recently utilized many metal complexes as chemotherapeutic agents for cancer treatment due to their ability to cross-link DNA and redox-mediated anticancer activity [38, 39]. Currently, several anticancer agents, such as NAMIA-A [40], cisplatin [41], and KP1019 [42], contain metal complexes (Fig. 4).

This review analyzes and discusses benzimidazoles’ hybridization and metal complexation as anticancer scaffolds since 2016. In addition to their structure–activity relationship (SAR), the molecular mechanisms underlying these benzimidazole hybrids and benzimidazole-metal complexes are discussed. The molecules in our review have shown improved activity and significantly lower IC_50_ values than others in different studies.

### Mechanism of action of benzimidazole hybrids

#### Induction of apoptosis

Apoptosis is a process of programmed cell death in living multicellular organisms. Moreover, removing unhealthy or old cells plays a significant role in maintaining health [43]. Apoptosis disturbance leads to many diseases, such as Alzheimer’s disease, Parkinson’s disease, Huntington’s disease, and cancer [44]. The extrinsic and intrinsic pathways are the main pathways involved in apoptosis [45]. The intrinsic pathway is activated by DNA damage and cytokine deprivation, while the immune system induces the extrinsic pathway. Executioner caspases (caspase-3, -6, and -7) are a class of cysteine proteases that cleave target proteins required for normal cell function. Activating caspases results in plasma membrane changes and the shrinkage of apoptotic cells, eventually leading to cell death [46]. The B-cell lymphoma-2 (BCL-2) protein family regulates the intrinsic pathway and includes proapoptotic effector proteins, proapoptotic BH3-only proteins, and antiapoptotic BCL-2 proteins. BCL-2 proteins inhibit apoptosis through the inhibition of the proapoptotic BCL-2 proteins, the BCL-2-associated X protein (BAX), and the BCL-2 homologous antagonist killer (BAK) [47]. BH3-only proteins inhibit the antiapoptotic BCL-2 protein [48].

**Fig. 5:**
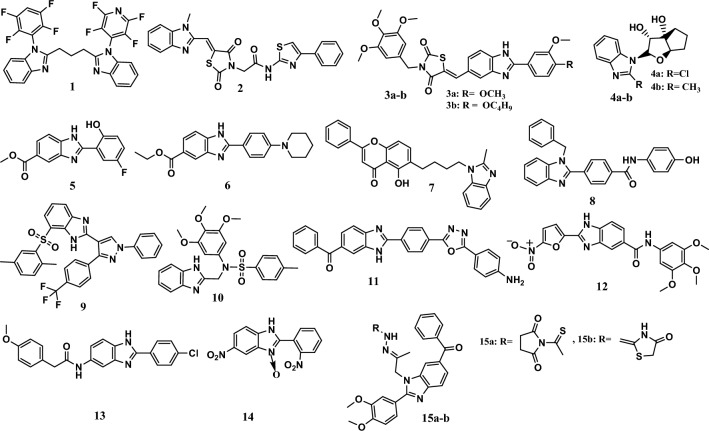
Structures of benzimidazole hybrids **1-15** induce apoptosis

Many benzimidazole derivatives display antiproliferative effects through the induction of the apoptosis pathway. For example, the calcein assay method showed that the fluoro aryl benzimidazole derivative 1 (Fig. 5) had potent anticancer activity, with IC_50_ values equal to 1.8 µM, 2 µM, 2.8 µM, and 7.8 µM against the HOS, G361, MCF-7, and K-562 cell lines, respectively. It was more potent than the reference drug roscovitine, which had an IC_50_ values equal to 24.3 µM, 22.4 µM, 42 µM, and 11 µM, respectively. According to flow cytometry analysis, its cytotoxic activity was attributed to the induction of apoptosis through the activation of caspase-3 and caspase-7 [49].

In addition, compound **2** was the most antiproliferative compound among the series of benzimidazole-thiazolidinedione hybrids (Fig. 5). It was more potent than 5-fluorouracil (5-FU) against the A549 lung cancer cell lines, with IC_50_ equal to 11.46 μM and 30.47 μM, respectively. Moreover, it had no toxic effect on the normal MCF10A cells. This compound had apoptotic effects through the collapse of the mitochondrial membrane potential and increasing the level of superoxide reactive oxygen species (ROS). In addition, it induced cell cycle arrest at the G2/M phase in a dose-dependent manner [50].

Moreover, compounds **3a-b** were other benzimidazole-containing thiazolidinedione derivatives (Fig. 5). These compounds showed potent antiproliferative activity, with IC_50_ values ranging from 0.096 μM to 0.32 μM against the HeLa and A549 cell lines; these values were comparable to those of nocodazole, which was used as a positive control, with IC_50_ values equal to 2.83 μM and 1.87 μM, respectively. The two compounds **3a-b** showed a safe profile against normal HeK-293T kidney cells. *In vitro* cell migration and wound healing assays of A549 cells showed that these compounds induced apoptosis by increasing intracellular ROS levels and decreasing the mitochondrial membrane potential. In addition, compound 3a-b caused a decrease in the mitochondrial membrane potential (DΨm) in A549 cells of 31.6% and 37.3%, respectively. Moreover, these compounds induced apoptosis in the early and late stages in A549 cells [51].

Compounds **4a-b** (Fig. 5) showed promising antiproliferative activities with IC_50_ values ranging from 0.08 μM to 0.31 μM against the MCF-7 and MDA-MB-231 cell lines. In comparison, doxorubicin, a standard drug, had IC_50_ values equal to 2.93 μM and 13.6 μM, respectively. The primary mechanism of action for compound **4a** was the induction of apoptosis, while compound **4b** arrested the cell cycle in the S phase. Molecular docking studies showed that compound **4a** binds to the minor groove region of DNA *via* three hydrogen bonds, and the binding energy was −7.6 kcal/mol. In contrast, compound **4b** exhibited different interactions, such as H-bonding by the hydroxyl group; its benzimidazole ring displayed π–anion interactions with the phosphate groups of the DC11 and DA18 nucleotides. Finally, the methyl group was bound to the DG16 base pair. In addition, the binding energy of **4b** was equal to −7.6 kcal/mol [52].

On the other hand, methyl 2-(5-fluoro-2-hydroxyphenyl)-1*H*-benzo[*d*]imidazole-5-carboxylate **5** (Fig. 5) displayed remarkable anticancer activity, with IC_50_ values equal to 0.39 μg/mL and 0.32 μg/mL against the HepG2 and Huh7 cell lines, respectively. This compound showed a safe profile against normal liver cells, with an IC_50_ = 40 μg/mL. It induces apoptosis through intrinsic and extrinsic pathways, cytochrome C activation, and caspase-3 and caspase-9 activation. In addition, it causes homeostasis through extreme ROS production [53].

Interestingly, ethyl 2-(4-(piperidine-1-yl)phenyl)-1*H*-benzo[*d*]imidazole-5-carboxylate **6** (Fig. 5) showed high selectivity against colorectal cancer (CRC) panel, with IC_50_ values equal to 16.82 μM and 20.11 μM against the HCT-116 and HT-29 cell lines, respectively. The colony formation assay showed 96.50% and 1.33% reduction in colony formation against HCT-116 and HT-29 cells, respectively, at 25 μM compared to 5-FU (103.5% and 163.7%, respectively). This compound induces apoptosis through sirtuin inhibition (SIRT1 and SIRT2 inhibition), resulting in DNA damage [54].

Moreover, a series of benzimidazoles linked to the natural flavone chrysin was synthesized and evaluated for anticancer activity. Among the synthesized hybrids, compound **7** (Fig. 5) had a remarkable antiproliferative activity, with an IC_50_ equal to 25.72 μM against MCF-7 cells, compared to 5-FU, which has an IC_50_ equal to 78.52 μM. The flow cytometry analysis showed that the compound induced apoptosis and arrested the cell cycle at the G0/G1 phase. Moreover, compound **7** showed promising tumor inhibition regression (TIR) of 71.9% according to an *in vivo* anticancer study at 10 mg/kg [55].

On the other hand, compound **8** (Fig. 5) showed moderate anticancer activity, with an IC_50_ equal to 7.01 μM against MCF-7 cells, while 5-FU, as a reference drug, had an IC_50_ value of 3.20 μM. Induction of apoptosis and cell cycle arrest at the G2/M and S phases are the primary mechanisms of action of this compound. Induction of apoptosis was proven by cell shrinkage, an increase in the level of ROS, and an increase in the mitochondrial membrane potential. In addition, compound **8** potently inhibited galectin-1, which was proven by Scarff–Bloom–Richardson (SBR) and fluorescence spectroscopy [56]. Interestingly, the MTT assay confirmed that compound **9** (Fig. 5) was a more potent cytotoxic agent than the reference drugs’ colchicine and combretastatin A-4 (CA-4). It exhibited IC_50_ values ranging from 0.15 μM to 0.33 μM against the A549, HeLa, HepG2, and MCF-7 cell lines. On the other hand, colchicine and CA-4 had IC_50_ values ranging from 0.22 μM to 0.44 μM and from 0.16 μM to 0.33 μM, respectively, against the same cell lines. Compound **9** exerted its anticancer activity by inducing apoptosis and cell cycle arrest at the G2/M phase. Furthermore, it inhibited tubulin polymerization with an IC_50_ equal to 1.52 μM [57].

Moreover, compound **10** was the most potent antiproliferative agent among a series of benzimidazole derivatives with sulfonamide moiety (Fig. 5). This compound showed more potent anticancer activity than 5-FU, with IC_50_ values ranging from 1.02 μM to 5.40 μM against the MGC-803, PC-3, and MCF-7 cell lines. Moreover, 5-FU had an IC_50_ ranging from 6.82 μM to 18.42 μM according to the MTT assay. It causes the induction of apoptosis and arrests the cell cycle at the G2/M phase. The SAR study indicated the importance of the 3,4,5-trimethoxy phenyl group at the *N*-1 position and tertiary sulfonyl groups for antiproliferative activity [58].

Moreover, hybrid **11** (Fig. 5) was the most potent compound among a series of 1,3,4-oxadiazole/benzimidazole hybrids. Hybrid **11** showed promising activity, with IC_50_ values equal to 1.87 μM and 5.67 μM against the MCF-7 and MDA-MB-231 cell lines, respectively. It was more potent than the erlotinib reference drug, which had an IC_50_ equal to 4.58 μM and 7.46 μM, respectively. Its antiproliferative activity was attributed to the induction of apoptosis by activating caspase-3 and caspase-9 and arresting the cell cycle at the G2/M phase. In addition, compound **11** showed a good safety profile against the WI-38 cells [59].

Conversely, compound **12** (Fig. 5) showed remarkable cytotoxic activity against the Jurkat E6.1 and HL-60 cell lines, with IC_50_ values of 2.95 µM and 12.12 µM, respectively. Its activity was comparable to that of the positive control doxorubicin, with IC_50_ values equal to 4.36 µM and 1.49 µM, respectively. According to the flow cytometry analysis, compound **12** displayed antiproliferative activity through the induction of apoptosis by inhibiting lysosome-induced autophagy or heme synthesis. Moreover, it showed an excellent safety profile against normal human leukocytes [60].

In addition, hybrid **13** (Fig. 5) displayed promising antiproliferative activity against HUVECs and HepG2 cells, with IC_50_ values equal to 1.47 μM and 2.57 μM, respectively. Also, it induced apoptosis and cell cycle arrest at the G1 phase. Moreover, this compound displayed robust antiangiogenic activity, attributed to the inhibition of vascular endothelial growth factor II (VEGFR-2) kinase, with an IC_50_ equal to 0.051 μM [61].

Compound **14** (Fig. 5) showed remarkable cytotoxic activity against T-47D cells under normoxic and hypoxic conditions. The IC_50_ values were 3.2 nM and 0.31 nM, respectively, comparable to the activity of the reference drug tirapazamine, which had IC_50_ values equal to 1.7 nM and 0.12 nM, respectively. Compound **14** promoted intercellular apoptotic pathway activity, which was proven by increased Bax protein expression. In addition, it potently inhibited NF-κB expression under hypoxic conditions [62].

Furthermore, compounds **15a-b** (Fig. 5), which contained a 6-benzoyl benzimidazole core, showed promising anticancer activity through the induction of apoptosis, cell cycle arrest at the G2/M phase, and cell accretion in the pre-G1 phase. Compounds **15a-b** had IC_50_ values of 1.62 µM and 1.44 µM, respectively, after 24 h of treatment against the HeLa cells [63].

**Fig. 6:**
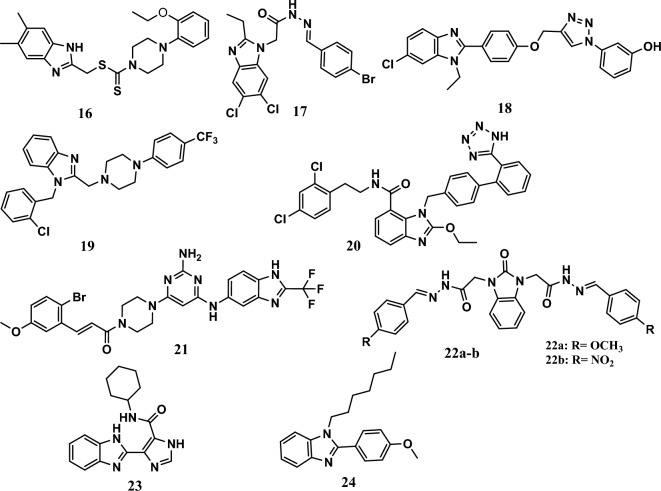
Structures of benzimidazole hybrids **16-24** induce apoptosis

On the other hand, compound **16** (Fig. 6) had potent antiproliferative activity, with an IC_50_ equal to 5.58 μg/mL against MCF-7 cells, which was comparable to the IC_50_ of doxorubicin, a standard drug, which has an IC_50_ of 4.1 μg/mL. The principal mechanism of action of compound **16** was the induction of apoptosis and cell cycle arrest at the G2/M phase. The SAR study showed that a di-thiocarbamate linker was essential for activity. A molecular docking study showed good binding of compound **16** to the Pin1 enzyme [64].

Furthermore, compound **17** (Fig. 6) showed good anticancer activity, with IC_50_ values equal to 17.8 μM and 10.2 μM against the MCF-7 and DU-145 cell lines, respectively. Moreover, 5-FU, a positive control, had IC_50_ values equal to 1.9 μM and 1.7 μM, respectively. Flow cytometric analysis revealed that the anticancer activity of compound **17** was attributed to the induction of apoptosis and cell cycle arrest at the G2/M phase. A SAR study proved that the bromine atom was essential for activity [65].

On the other hand, among the benzimidazole-triazole hybrids tested, compound **18** (Fig. 6) showed remarkable anticancer activity, with an IC_50_ equal to 0.63 μM, 0.99 μM, 1.3 μM, and 0.94 μM against the A549, NCI-H460, MCF-7, and MDA-MB-231 cell lines, respectively, according to the MTT assay results. In comparison, 5-FU had IC_50_ values equal to 1.69 μM, 3.20 μM, 2.80 μM, and 0.79 μM, respectively. The primary mechanism of its anticancer activity was the induction of apoptosis, cell cycle arrest at the sub-G1 phase, and increased ROS levels, which decreased the mitochondrial membrane potential [24].

Compound **19** (Fig. 6) displayed remarkable cytotoxic activity against the A549 and MCF-7 cancer cell lines, with IC_50_ values equal to 5.4 μM and 4.2 μM, respectively. It induces apoptosis through the cleavage of poly (ADP-ribose) polymerase 1 (PARP-1) and activation of caspase-7 [66].

On the other hand, compound **20** (Fig. 6) had antiproliferative effects on A549 and H1299 cell lines, with IC_50_ values equal to 20.39 μM and 21.76 μM, respectively, while the reference drug MLN4924 had IC_50_ values of 0.63 μM and 0.49 μM, respectively. In addition, it was safer than MLN4924 against normal cell lines, 16HBE and BEAS-2B, which had IC_50_ values equal to 76.06 μM and 79.35 μM, respectively, compared to MLN4924 (IC_50_ values = 4.03 μM and 1.22 μM, respectively). According to the annexin-V/PI double staining and SA-b-gal staining results, apoptosis was induced by hybrid **20**. An *in vivo* study of the mice showed that the growth of A549 xenograft tumors was inhibited through injection at a dose of 30 mg/kg, and a potent effect was observed through oral administration of the drug at a dose of 60 mg/kg. A molecular docking study demonstrated the greater affinity of compound **20** for the ATP pocket. In addition, an electron-withdrawing group (Cl atom) was essential for its activity [67].

Moreover, compound **21** (Fig. 6) was the most active compound among a series of benzimidazole-pyrimidine hybrids. The compound **21** exhibited IC_50_ values ranging from 2.21 µM to 7.29 µM, while nocodazole, a positive control, had IC_50_ values ranging from 1.96 µM to 3.48 µM against the A549, PC-3, HeLa, and MDA-MB-231 cell lines. In addition, it had no significant inhibitory effect on the noncancerous cell line of human pulmonary epithelial cells (L132) (IC_50_ = 69.25 μM). Interestingly, it induced apoptosis in A549 cells by increasing ROS levels and decreasing the mitochondrial membrane potential, and it exerted anti-colonigenic effects. Moreover, it inhibited tubulin polymerization with an IC_50_ value of 5.72 µM, and it caused cell cycle arrest at G2/M. In addition, compound **21** exhibited an increased affinity for the colchicine-binding site, and the cinnamide ring was forcefully hidden into the hydrophobic pocket colchicine-binding site of α,β-tubulin (PDB ID: 1SA0) [68].

Compounds **22a-b** (Fig. 6) displayed antiproliferative activity against HeLa and HCT-116 cells, with IC_50_ values ranging from 29.5 µM to 57.1 µM. Interestingly, the compounds showed no anticancer activity against HEK-293 normal cells. These compounds exert their anticancer effects by inducing apoptosis, shrinkage & loss of the shape of the nuclei, and increasing nuclear condensation, according to the results of 4′,6-diamidino-2-phenylindole (DAPI) staining [69].

Compound **23** (Fig. 6) showed anticancer activity like cisplatin, and it was more potent than 5-FU against human triple-negative breast cancer (TNBC) cell lines MDA-MB-231 and MDA-MB-468 with IC_50_ values equal to 6.03 μM and 8.3 μM, respectively, while the IC_50_ values of cisplatin were 3.07 μM and 6.71 μM, respectively. In contrast, 5-FU had IC_50_ values equal to 26.73 μM and >200 μM. Compound **23** exerted its antiproliferative effect through the induction of apoptosis by decreasing the membrane potential of mitochondria and causing DNA damage. In addition, the compound arrested the cell cycle at the S phase [70].

Recently, compound **24** (Fig. 6) was the most potent antiproliferative agent against the MDA-MB-231 cells among *N*‑Alkylated-2-(substituted phenyl)‑1*H*‑benzimidazole derivatives. It had an IC_50_ equal to 16.38 μM compared to camptothecin (IC_50_ = 0.41 μM). The cytotoxic effect of compound **24** could be attributed to its lipophilicity. This compound might induce apoptosis through chromatin condensation, abnormal DNA replication, and abnormal mitosis [71].

#### EGFR inhibition

The EGFR is an ErbB family member that plays a critical role in tumor progression and regulation of the mitogen-activated protein kinase (MAPK) pathway. The MAPK pathway regulates angiogenesis, impacting cell division and differentiation. Overexpression of the EGFR gene and EGFR tyrosine kinase (EGFR TK) domain mutations have been linked to the development and progression of several cancers, including lung, colorectal, breast, brain, and pancreatic [72, 73].

**Fig. 7:**
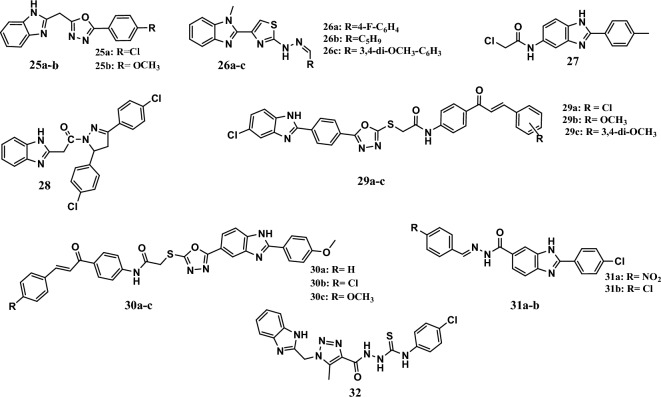
Structures of benzimidazole hybrids **25-32** as EGFR inhibitors

Among the series of benzimidazole-linked oxadiazole derivatives, compounds **25a-b** (Fig. 7) were the most active compounds, with IC_50_ values ranging from 0.13 μM to 15.2 μM against the MCF-7, HaCaT, MDA-MB231, HepG2, and A549 cell lines. Moreover, 5-FU, a reference drug, had an IC_50_ ranging from 1.16 μM to 7.12 μM. Moreover, these compounds showed promising inhibitory effects on EGFR and ErbB2, with IC_50_ values ranging from 0.08 μM to 0.91 μM, while the gefitinib reference drug had IC_50_ values of 0.011 μM and 0.024 μM against EGFR and erbB2, respectively. The compounds showed apoptotic activities accompanied by cell cycle arrest at the G2/M phase, according to FACS analysis. A molecular docking study revealed the potent binding of compounds **25a-b** to the ATP-binding site of the kinase domain of the EGFR receptor (PDB 1M17) [30].

Thiazole/benzimidazole hybrids **26a-c** (Fig. 7) had comparable antiproliferative activities to erlotinib against MCF-7 cells. Their IC_50_ values were 5.96 μM, 6.30 μM, and 7.56 µM, respectively, while the IC_50_ of erlotinib was 4.15 μM. All the compounds showed significant effects as EGFR TK inhibitors, with IC_50_ values of 253.74 nM, 109.71 nM, and 208.09 nM, respectively, compared to the erlotinib IC_50_, which was equal to 152.59 nM. Moreover, compound **26b** induced apoptosis by decreasing Bcl-2 and PARP-1 enzyme levels and increasing caspase-3, p53, and BAX levels. In addition, hybrids **26a-c** exhibited cell cycle arrest at the G2/M phase with a necrosis effect equal to 2.17%. The presence of fluorine as an electron-withdrawing group in compound **26a** increased the activity. It enhances the carbon‒fluorine bond energy and increases the lipophilic character of the compound, which might improve cell membrane permeability. A docking study showed promising binding affinity at the EGFR pocket [74].

Moreover, compound **27** (Fig. 7) had moderate EGFR inhibitory activity, with an IC_50_ equal to 1.93 μM, comparable to the positive control gefitinib, which had an IC_50_ equal to 4.2 nM. An *in vitro* kinase assay showed that compound **27** at 50 μM exhibited 88% EGFR inhibitory activity. A molecular docking study revealed the good binding affinity of compound **27** at the ATP-binding site of EGFR kinase (PDB: 2J6M) through binding with the amino acid MET793 through a hydrogen bond [75].

In addition, compound **28** (Fig. 7) was the most potent antiproliferative agent among the series of benzimidazoles linked to pyrazoles. Compound **28** exhibited an IC_50_ ranging from 2.2 µM to 11.9 µM against the MCF-7, MDA-MB231, and A549 cell lines compared to that of the 5-FU reference drug (IC_50_ ranging from 1.16 µM to 7.12 µM). It induces cell cycle arrest at the G2/M phase according to the FACS analysis results. Furthermore, it had remarkable EGFR inhibitory activity, with an IC_50_ equal to 0.970 µM, compared to the IC_50_ of the gefitinib reference drug (IC_50_ = 0.011 µM). The molecular docking of compound **28** showed good binding at the ATP pocket of the EGFR kinase domain (PDB 1M17) with five hydrogen bonds and two π-interactions. In addition, it had a binding energy equal to −34.581 kcal/mol, which was comparable to erlotinib's binding energy (−30.96 kcal/mol) [76].

Recently, a series of 1,3,4‐oxadiazole‐chalcone/benzimidazole hybrids were synthesized as anticancer agents. Among them, compounds **29a-c** (Fig. 7) had a potent antiproliferative activity with IC_50_ values ranging from 0.80 µM to 2.10 µM against A‐549, HT‐29, MCF‐7, and Panc‐1 cell lines compared to doxorubicin reference drug (IC_50_ values ranging from 0.90 µM to 1.41 µM). Compound **29b** was the most potent compound, with IC_50_ values ranging from 0.8 µM to 1.30 µM. Compounds **29a-c** showed moderate EGFR inhibition activity with IC_50_ ranging from 1.80 μM to 3.60 μM comparable to the erlotinib reference drug (IC_50_ = 0.06 μM). At the same time, compounds **29a-c** showed Braf inhibitory activity with IC_50_ values ranging from 4.6 μM to 8.9 μM comparable to erlotinib (IC_50_ = 0.05 μM). Moreover, compounds **29a-b** arrested the cell cycle at the G1/S Phase and Pre-G1. In addition, compound **29a-b** had a higher late apoptosis percentage than early apoptosis. Compounds **29a-c** revealed binding affinity at ATP-binding sites of EGFR (PDB ID: 1M17) with binding score values ranging from −8.40 to −8.66 kcal/mol comparable to erlotinib (binding score = −7.81 kcal/mol) [77]. Moreover, compounds **30a-c** (Fig. 7) showed potent antiproliferative activity against Panc-1, A549, MCF-7, and HT-29 cell lines with IC_50_ ranging from 0.95 μM to 1.85 μM, while doxorubicin reference drug had an IC_50_ ranging from 0.90 μM to 1.41 μM. In addition, compound **30b** showed antiproliferative activity against LOX-IMVI cells with an IC_50_ of 0.80 µM, while the staurosporine reference drug exhibited an IC_50_ equal to 7.10 µM. Compounds **30a-c** exhibited potent EGFR inhibitory activity with IC_50_ values ranging from of 0.55 µM to 0.9 µM comparable to erlotinib (IC_50_ = 0.08 µM). In addition, they showed moderate BRAF inhibitory activity with IC_50_ values ranging from 0.90 µM to 1.00 µM, while erlotinib had an IC_50_ equal to 0.06 µM. Compounds **30a-c** showed anticancer activity through induction of apoptosis by intrinsic and extrinsic pathways, which was proven by overexpression of caspase-3, -8, and- 9 with fold change equal to the doxorubicin reference drug, increased Cytochrome C levels with fold change 11–14 times compared to controlled and decreased antiapoptotic protein BCL-2 and increase apoptotic BAX protein. Compound **30a** arrested cell cycle in MCF-7 at the G1/S phase. A Docking study indicated that compounds **30a-b** showed binding scores ranging from −10.6 to −10.4 kcal/mol compared to erlotinib (binding score = −9.7 kcal/mol) at ATP-binding sites of EGFR (PDB ID: 1M17), while Molecular Docking Studies on BRAF (PDB ID: 3OG7) showed Binding scores ranging from −9.8 to −9.9 kcal/mol while Vemurafenib and Erlotinib-positive control with docking scores equal to −9.6 and −9.5 kcal/mol, respectively. A Molecular dynamic stimulation validated the docking results and showed high stability inside the active sites of both EGFR and BRAF enzymes [78].

Furthermore, among a series of benzimidazole-hydrazone derivatives, compounds **31a-b** (Fig. 7) displayed promising and broad-spectrum anticancer activity without selectivity with GI_50_ values ranging from 0.420 µM to 8.99 µM. Moreover, compounds **31a-b** exhibited potent EGFR inhibitory activity with IC_50_ values equal to 0.11 µM and 0.09 µM, while the erlotinib had an IC_50_ equal to 0.08 µM. In addition, compounds **31a-b** displayed potent BRAF inhibitory activity with IC_50_ values of 0.31 µM and 0.20 µM, respectively. At the same time, erlotinib and vemurafenib positive controls had IC_50_ values equal to 0.06 µM and 0.03 µM, respectively. Furthermore, the compounds upregulated caspase-3, caspase-8, and Bax and downregulated antiapoptotic Bcl-2 protein [79].

In addition, compound **32** (Fig. 7) was the most potent compound among a series of benzimidazole-triazole hybrids. The compound showed promising anticancer activity with IC_50_ values ranging from 3.87 μM to 8.34 μM against HCT-116, HepG2, MCF-7, and HeLa cancer cell lines comparable to doxorubicin reference drug (IC_50_ values ranging from 4.17 μM to 5.57 μM). Compound **32** displayed promising EGFR inhibitory activity with an IC_50_ equal to 0.086 µM, while gefitinib-positive control had an IC_50_ of 0.052 µM. Furthermore, it showed potent Topo I inhibitory activity with an IC_50_ of 2.52 µM comparable to doxorubicin (IC_50_ = 3.62 µM). Compound **32** arrested HepG2 cells at the G1/S phase and induced apoptosis at the late stage with DNA intercalation activity with an IC_50_ value of 33.17 µM like doxorubicin (IC_50_ = 31.54 µM). Moreover, compound **32** showed a safe profile on WI-38 normal cells [80].

#### BRAF inhibition

**Fig. 8:**
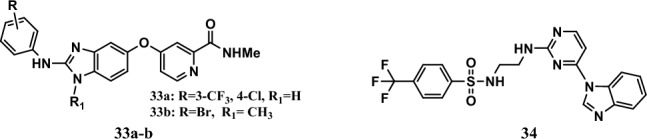
Structures of benzimidazole hybrids **33-34**

The BRAF gene encodes a protein found on chromosome 7. It regulates cell proliferation and apoptosis in response to extracellular stimuli, such as growth factors, hormones, cytokines, and other environmental stresses. BRAF mutation causes cancer cell growth by activating the MAPK pathway. Two drugs, vemurafenib and dabrafenib, were approved by the Food and Drug Administration (FDA) as BRAF inhibitors [73, 81]. In addition, two compounds, **33a-b** (Fig. 8), were prepared as sorafenib analogs by cyclization of the urea chain, and Novartis reported these two compounds as BRAF kinase inhibitors with IC_50_ values equal to 0.28 μM and 0.002 μM, respectively [82].

In addition, compound **34** (Fig. 8) was the most potent compound among a series of 4-(1*H*-benzo[*d*]imidazol-1-yl)pyrimidine-2-amine hybrids linked to sulfonamide. The compound showed more potent activity than the sorafenib reference drug with IC_50_ values equal to 2.02 μM and 1.85 μM against the SK-MEL-5 and A375 cell lines, respectively, while sorafenib had IC_50_ values of 9.22 μM and 5.25 μM, respectively. In addition, the compound significantly inhibited BRAFV600E by 98.75% at a concentration of 1 μM, which was comparable to that of sorafenib (84.57%). In addition, it showed potent activity against the BRAF^V600E^ protein, with an IC_50_ equal to 0.49 μM. A molecular docking study revealed the high affinity of compound **34** for the binding site of the BRAF^V600E^ protein, in which the binding energy was equal to –25.86 kcal/mol, compared to vemurafenib, which is a reference drug with a binding energy equal to −31.41 kcal/mol [31].

#### Induction of ferroptosis

Ferroptosis is a recently discovered type of programmed cell death characterized by the accumulation of a large amount of iron, which causes lipid peroxidation after the Fenton reaction, leading to intracellular oxidative stress [83]. Small molecules such as sulfasalazine (SAS), RSL-3, and erastin can induce ferroptosis [84]. Ferroptosis may be induced through different pathways, either through glutathione peroxidase-4 (GPX4) through suppression of the synthesis of GPX4 or through a GPX4-independent pathway [85, 86]. Morphologically, ferroptosis is distinct from other programmed cell death processes and involves mainly mitochondrial morphology, as indicated by shrinkage of mitochondria with a low amount of mitochondrial cristae or without mitochondrial cristae, a condensed cell membrane density, and rupture of the mitochondrial membrane [87].

Compound **35** showed promising anticancer activity, with an IC_50_ of 1.26 μM against HT1080 cells. In comparison, the Erastine reference drug exhibited an IC_50_ equal to 1.52 μM. The compound acted as a ferroptosis inducer in different ways. For example, the first one involved measuring the amount of lipid ROS accumulation, which was equal to that of erastin. In addition, morphological changes, such as mitochondrial shrinkage, are related to ferroptosis morphology. Moreover, the ferroptosis inhibitors Fer-1, DFO, and Trolox protected HT1080 cells from ferroptosis but not from apoptosis or necrosis. Ferroptosis was induced by inhibiting the Xc−GSH-GPX4 system in treated HT1080 cells. Moreover, it showed more advantages than did erastin in terms of metabolic stability. It was safe to use *in vivo* and had a safe profile [25].

#### Inhibition of tubulin polymerization

Microtubules, which consist of α- and β-tubulin heterodimers, are fundamental for an assortment of basic cell processes, such as cell division, maintenance of cell shape, motility, cell flagellin, intracellular vehicles, and isolation of chromosomes during mitosis. Hence, inhibition of tubulin polymerization helps prevent mitosis and division of cancer cells [88].

**Fig. 9:**
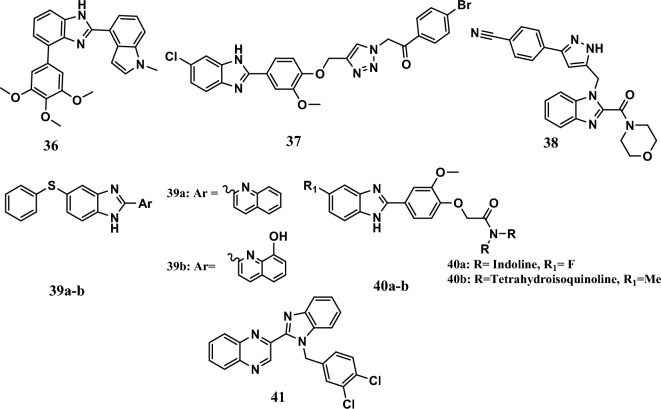
Structures of benzimidazole hybrids **36-41**

Some benzimidazoles inhibited tubulin polymerization. For example, compound **36** (Fig. 9) displayed significant anticancer activity among indole and benzimidazole hybrids as tubulin polymerization inhibitors. The compound inhibited the colony formation of paclitaxel-resistant cell lines A2780/T and its parental cell line A2780/S, with IC_50_ values equal to 6.20 nM and 9.70 nM, respectively. In comparison, 100 nM paclitaxel did not stop colony formation. An *in vivo* study showed that the compound exhibited remarkable antitumor effects in a melanoma tumor model, with tumor growth inhibition rates of 78.70% (15 mg/kg) and 84.32% (30 mg/kg) in a dose-dependent manner [89].

On the other hand, compound **37** (Fig. 9) was the most potent compound among a series of 1,2,3-triazolo-2-aryl-linked benzimidazole derivatives. It had an IC_50_ of 4.17 µM, while 5-FU had an IC_50_ of 14.64 µM against SK-Mel-28 cells. The compound showed a remarkable safety profile against the normal human cell line BEAS-2B, with an IC_50_ >100 µM. It inhibited tubulin polymerization with an IC_50_ equal to 5.65 μM. This result agreed with the docking study. The compound also displayed different mechanisms of action, such as preventing colony formation, inducing apoptosis, interacting with CT-DNA, increasing the level of ROS, and damaging DNA by decreasing the mitochondrial membrane potential [90].

Moreover, compound **38** (Fig. 9) displayed potent anticancer activity with IC_50_ values equal to 0.042 μM, 0.61 μM, and 0.76 μM against MCF-7, PC-3, and A549 cell lines, respectively, compared to IC_50_ of etoposide standard drug equal to 2.11 μM, 2.39 μM, and 3.08 μM, respectively. Interestingly, the compound inhibited tubulin polymerization with an IC_50_ of 0.35 μM. It was more effective than the CA-4 standard drug (IC_50_ = 1.12 μM). A docking study showed that the compound had good binding affinity for α,β-tubulin (PDB ID-1SA0), with a binding energy equal to −9.69 kcal/mol. In addition, the compound was found to follow the Lipinski, Muegge, Ghose, Egan, and Veber rules without any deviation [32].

Recently, compounds **39a-b** (Fig. 9) were the most potent compounds in a series of benzimidazole derivatives designed as tubulin polymerization inhibitors. Compounds **39a-b** showed anticancer activity against the A549 cell line with IC_50_ values of 2.90 µM and 2.33 µM, respectively, and with selectivity indexes of 5.81 and 5.20, respectively ,comparable to colchicine, nocodazole, and paclitaxel as positive controls with IC_50_ equal of 2.3 µM, 1.90 µM, and 1.39 µM, respectively. Compounds **39a-b** showed anticancer activity through stabilizing the microtubule network by upregulation of DeY-α-tubulin and downregulation of Ac-α-tubulin expressions in cancer cells. Compounds **39a-b** induced cell cycle arrest in the G_2_/M phase with 22% cells and induced apoptosis at the early phase. The molecular docking study revealed that the two compounds established higher binding affinity for the nocodazole binding site (NBS) (PDB: 5CA1) with binding scores equal to −9.872 kcal/mol and −8.494 kcal/mol, respectively, while their binding scores in the colchicine-binding site (CBS) (PDB: 4O2B) were −7.74 kcal/mol and −9.757 kcal/mol [91].

Moreover, compounds **40a-b** (Fig. 9) were the most potent compounds from a series of benzimidazole-based carboxamide derivatives. Compounds **40a-b** showed high cytotoxic activity specificity against SKMel-28 cells with IC_50_ values equal to 2.69 µM and 2.55 µM, respectively, comparable to nocodazole as reference drug (IC_50_ = 8.59 µM). Furthermore, at a dosage of 5.05 μM, compound **40a** reduced tubulin polymerization by 52.4%. In addition, the compound **40a** arrested cell cycle at the G2/M phase in dose-dependent manner. A molecular modeling study revealed that compound **40a** has a good fit to the active pocket of the α/β-tubulin receptor (PDB ID: 3E22), and it forms stable hydrogen bonds with amino acid residues [92].

Furthermore, compound **41** (Fig. 9) was the most potent compound in between a series of quinoxaline-benzimidazole hybrids. Compound **41** showed potent cytotoxic activity against A549 cells with an IC_50_ equal to 4.37 μM. It inhibited tubulin polymerization with an IC_50_ equal to < 2.19 µM. A molecular docking study proved that **41** has a potent binding affinity at CBS of α/β- tubulin with a binding energy of −45.139 kcal/mol and with potent protein–ligand interactions. In addition, the compound induced apoptosis in A549 cells and arrested the cell cycle at the G2/M phase [93].

#### Interactions with DNA

**Fig. 10:**
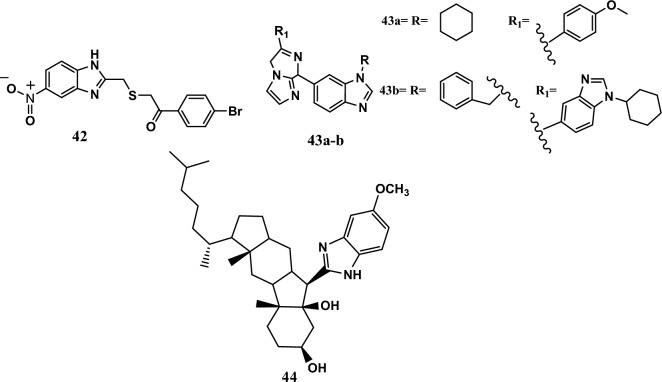
Structures of benzimidazole hybrids **42-44**

Currently, preventing DNA replication is a valuable pathway for treating cancer. Drug interactions with DNA block DNA synthesis. Drug‒DNA interactions can occur through three binding modes: intercalation, electrostatic binding, and minor or major groove interactions. Small molecules bind to DNA by forming hydrogen or van der Waals bonds [94]. Compound **42** (Fig. 10) was an example of a drug that interacted with DNA and showed potent anticancer activity, with IC_50_ values equal to 2.01 μM, 1.78 μM and 1.55 μM against HepG2, HCT-116, and MCF-7 cancer cell lines, respectively, according to the MTT assay. Moreover, 5-FU, a standard drug, exhibited IC_50_ values equal to 5.30 μM, 4.00 μM, and 4.30 μM for the same cell lines. The compound showed a safe profile against the WI-38 human normal cell line, with an IC_50_ equal to 31.47 μM, compared to 5-FU, which has an IC_50_ equal to 5.73 μM. In addition, the methyl green/DNA displacement assay showed that compound **42** had a strong DNA-binding affinity with an IC_50_ equal to 34.56 μM, comparable to that of doxorubicin as a reference drug (IC_50_ = 32.35 μM). According to the SAR study, 4-bromo substitution at the phenacyl moiety increases lipophilicity, increasing anticancer activity [95].

Furthermore, two compounds, **43a-b** (Fig. 10), exhibited the most potent activity among a series of imidazo[1,2-*a*]pyrazine-benzimidazole hybrids. The SRB assay evaluated the *in vitro* cytotoxic effects of compounds **43a-b** on 60 cell lines. Compounds **43a-b** had MG_MID GI_50_ values equal to 2.10 μM and 2.23 μM, respectively. According to the ethidium bromide assay, the main mechanism of action of these agents was through interactions with DNA and bovine serum albumin. Moreover, molecular docking results indicated a minimum binding energy of −10.70 kcal/mol and −9.30 kcal/mol in the DNA-binding site [96].

In addition, compound **44** (Fig. 10) interacted with the calf thymus DNA (ct-DNA) of tumor cells, and its binding was measured *via* different methods, such as fluorescence spectroscopic techniques, UV‒Vis spectroscopy, and KI quenching experiments. The compound from B-norcholesteryl and benzimidazole had the best activity among the hybrids. The IC_50_ ranged from 7.90 μM to 20.10 μM against the HeLa, MCF-7, T-47D, and SKOV3 cell lines. The SAR study proved that the electron-donating group smoothed the insertion of the molecule into DNA [97].

#### Topoisomerase II inhibition

**Fig. 11:**
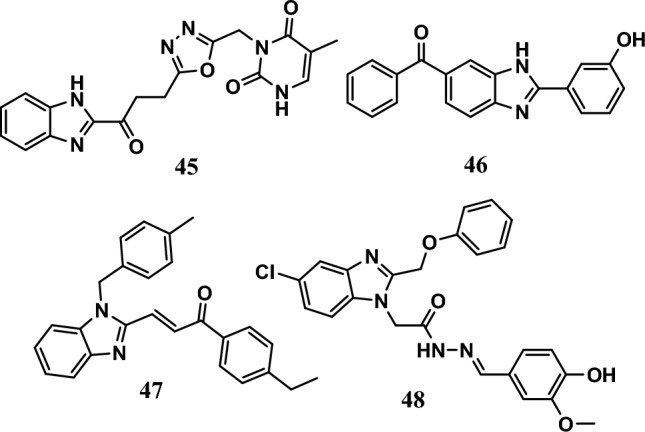
Structures of benzimidazole hybrids **45-48**

Selective TOPO II inhibitors are important chemotherapeutic agents that enhance cell cycle arrest and induce apoptosis by suppressing DNA replication and transcription [98]. Compound **45** (Fig. 11) acted as a TOPO II inhibitor by binding to the binding site of the TOPO enzyme. It had potent antiproliferative effects, with GI_50_ values equal to 0.09 µM and 0.35 µM against the SNB-75 and COLO 205 cell lines, respectively. Moreover, it showed moderate selectivity toward prostate cancer cell lines, with a selectivity index of 3.66. SAR studies showed that benzimidazole derivatives containing a 1,3,4-oxadiazole ring had greater anticancer activity than benzimidazoles with other heterocyclic rings in this study, such as thiadiazole, triazolo-thiadiazines, and triazolo-thiadiazoles [99].

Moreover, compound **46** (Fig. 11) was the most active hybrid among a series of 2-phenyl benzimidazoles. The IC_50_ values were 4.47 μg/mL, 4.68 μg/mL, and 5.50 μg/mL against the A549, MDA-MB-231, and PC-3 cell lines, respectively. Its activity was comparable to that of the standard camptothecin, which had IC_50_ values equal to 0.2 μg/mL, 0.47 μg/mL, and 0.87 μg/mL. Moreover, its main mechanism of action involves binding with TOPO and DNA [100].

Moreover, benzimidazole-chalcone hybrid **47** (Fig. 11) showed potent TOPO inhibition, for which the inhibition rate was 108.6%, compared to the etoposide positive control (73.6%) and chalcone (12.5%). Compound **47** had IC_50_ values ranging from 3.70 μM to 6.50 μM against the A549, HePG2, MG63, and LNCaP cell lines. It was more potent than etoposide, which had IC_50_ ranging from 5.20 μM to 13.30 μM, while chalcone exhibited IC_50_ values ranging from 52.40 μM to >100 μM. It exerts its antiproliferative effect by inhibiting TOPO II-mediated DNA relaxation, cell migration, and colony formation [101].

In addition, compound **48** (Fig. 11) displayed remarkable anticancer activity through different mechanisms: inhibition of TOPO II with an IC_50_ equal to 6.72 µM, while the standard drug staurosporine had an IC_50_ of 4.64 µM, arresting the cell cycle at the S phase in HepG2 cancer cells and inducing apoptosis. Furthermore, the compound induced apoptosis by increasing caspase-3, caspase-8, and caspase-9 levels and ROS levels and decreasing antiapoptotic Bcl-2 and IAP levels [102].

#### Antiestrogenic activity

**Fig. 12:**
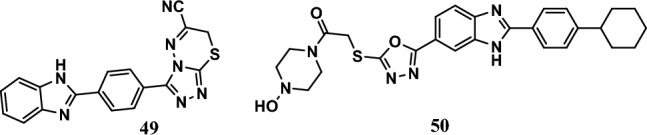
Structures of benzimidazole hybrids **49-50**

Breast cancer is dependent on estrogen or progesterone hormones. Estrogen synthesis occurs mainly in breast tissues that contain the aromatase enzyme, which is responsible for converting the enone ring of the androgen enzyme to testosterone to phenol and for the synthesis of estrogen [103]. Therefore, there is a continuous need for anticancer agents with antiestrogenic activity. Compound **49** (Fig. 12), which has a cyano group, was the most potent among the benzimidazole-triazolothiadiazine hybrids. This compound showed potent antiproliferative activity with an IC_50_ of 0.016 µM against MCF-7 cells. Cisplatin, as a positive control, had an IC_50_ of 0.020 µM. Moreover, compound **49** displayed significant aromatase inhibitory activity, with an IC_50_ of 0.032 µM, compared to letrozole (IC_50_ = 0.024 µM) [104].

On the other hand, compound **50** (Fig. 12) displayed promising anticancer activity against the MCF-7 and HepG2 cell lines, with IC_50_ values equal to 5.17 µM and 5.99 µM, respectively. Moreover, the doxorubicin reference drug had IC_50_ values equal to 10.53 µM and 14.28 µM, respectively. In addition, the aromatase enzyme was inhibited by compound **50** at an IC_50_ equal to 2.31 µM, whereas letrozole inhibited aromatase at an IC_50_ equal to 0.032 µM. Compound **50** stably interacted with the aromatase enzyme at the active site by forming a hydrogen bond. In addition, compound **50** obeyed the rules of Lipinski [105].

#### Induction of cell cycle arrest

**Fig. 13:**
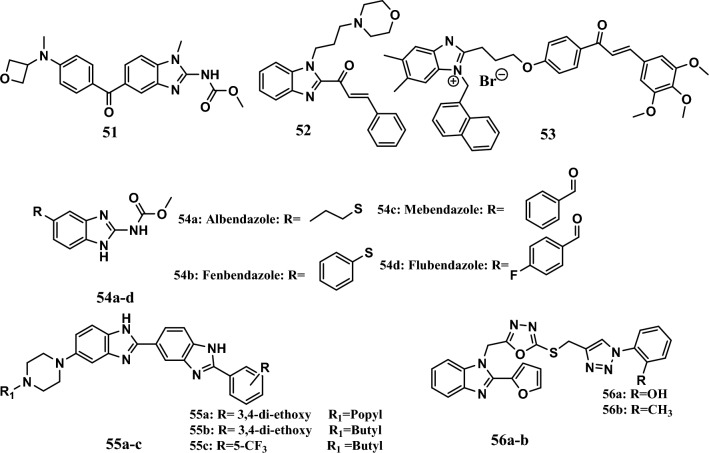
Structures of benzimidazole hybrids **51-56**

Cancer development is accompanied by upregulation of the cell cycle, which is divided into four phases: G1, S, G2, and M [106]. The cell cycle controls the replication of genomic DNA and separation between daughter cells, which occurs during cell cycle phases in eukaryotic cells [107]. Many benzimidazoles cause cell cycle arrest. For example, carbamate compound **51** (Fig. 13) showed promising anticancer activity, with EC_50_ values ranging from 0.9 µM to 2.4 µM against the H157, Calu1, A549, PC-3, PC3MLN4, and SKOV3 cell lines. In comparison, mebendazole, a reference drug, exhibited an EC_50_ ranging from 0.5 µM to 3.5 µM against the same cell lines. It causes cell cycle arrest at the G2/M phase and disrupts tubulin dynamics [108].

Moreover, compound **52** (Fig. 13) showed promising antiproliferative activity, with IC_50_ values equal to 8.91 μM, 10.93 μM, and 10.67 μM against the MCF-7, HEP-G2, and OVCAR-3 cell lines, respectively, compared to cisplatin, which exhibited IC_50_ values equal to 11.70 μM, 3.97 μM, and 16.04 μM, respectively. SAR studies showed that the* N*-substitution of benzimidazole with a hydrocarbon spacer linked to nitrogen was essential for activity. According to the flow cytometry analysis, compound **52** arrested the cell cycle at the S or G2/M phase [109].

Interestingly, chalcone-benzimidazolium derivatives with trimethoxy phenyl rings were synthesized as anticancer scaffolds. Compound **53** (Fig. 13) was the most active compound in this series, with IC_50_ values ranging from 0.83 μM to 7.97 μM against the HL-60, SMMC7721, A549, MCF-7, and SW480 cell lines. Its activity was comparable to that of cisplatin, which was used as a positive control (the IC_50_ ranged from 2.11 μM to 17.43 μM). Cell cycle analysis of SMMC7721 cells treated with compound **53** indicated that this compound has antiproliferative effects through the induction of apoptosis and cell cycle arrest at the G1 phase [110].

Furthermore, the anticancer effects of the anthelmintic drugs albendazole, mebendazole, fenbendazole, and flubendazole **54a-d** (Fig. 13) on TNBC cells, such as MDA-MB231 and radiotherapy-resistant (RT-R) MDA-MB-231 cells, were studied. It was demonstrated that the compounds at 0.5 μM and 1 μM inhibited cell cycle progression in the G2/M phase by increasing cyclin B1 protein levels, and they inhibited tubulin polymerization. Furthermore, at these concentrations, all the compounds were safe for treating normal breast epithelial cells [111].

In addition, Fenbendazole **54c** was evaluated for its anticancer activity against five melanoma cell lines: the UCDK9M3, UCDK9M4, UCDK9M5, KMeC, and LmeC cell lines. Fenbendazole **54c** had IC_50_ values ranging from 0.47 μM to 4.10 μM. Moreover, Fenbendazole arrested the cell cycle at the G2/M phase with mitotic slippage in all melanoma cell lines except UCDK9M3 cells, which showed no mitotic slippage [112].

Moreover, the 1*H*-benzo[*d*]imidazole/piperazine hybrids **55a-c** (Fig. 13) exhibited remarkable activity against 60 NCI cell lines, with GI_50_ values ranging from 0.2 μM to 3.68 μM. All the compounds exhibited toxic effects on HEK and NIH3T3 with IC_50_ values ranging from 2.3 µM to 14.4 µM according to the MTT assay. The test compounds arrested the cell cycle at G2/M. In addition, the molecular docking study showed a binding affinity with TOPO I equal to −5.45 kcal/mol, −5.42 kcal/mol, and −5.51kcal/mol, which was comparable to that of camptothecin standard drug (binding affinity = −6.001 kcal/mol) [113].

However, compounds **56a-b** (Fig. 13) were the most potent compounds among the benzimidazole-1,3,4-oxadiazole derivatives. The compounds showed promising IC_50_ values ranging from 1.15 μM to 5.27 μM against the A549, SKOV3, and MDA-MB231 cell lines compared to the positive control 5-FU (IC_50_ range = 10.65 μM**-**15.48 μM). A mechanistic study showed that compounds **56a-b** arrested the cell cycle at the G1 phase in MDA-MB-231 cells. Compound **56a** arrested the cell cycle at the G2 phase in the A549 and SKOV3 cell lines, while compound **56b** arrested the cell cycle at the G1 and S phases. In addition, these two compounds induced apoptosis without causing necrosis in the A549, SKOV3, or MDA-MB231 cell lines [114].

#### Glutathione S-transferase enzyme inhibition

Glutathione *S*-transferase enzymes play vital roles in cancer progression, as they aid in cancer cell proliferation and drug resistance [115]. Therefore, its inhibition may have a significant role in cancer treatment. Compounds **57a-b** showed potent inhibition of the glutathione *S*-transferase enzyme, with IC_50_ values equal to 0.0017 mg/mL and 0.00067 mg/mL, respectively, compared to ethacrynic acid, which has an IC_50_ equal to 0.0055 mg/mL, as a reference drug. In addition, compounds **57a-b** displayed moderate activity against MCF-7 and HCT cell lines, with IC_50_ values ranging from 7.50 μg/mL to 33.80 μg/mL. The doxorubicin reference drug had an IC_50_ equal to 1.20 μg/mL and 1.40 μg/mL, respectively. The SAR study showed that the isoxazole ring in **57a** and the 2-methyl-3-acetyl-pyridine moiety in **57b** increased the activity three and ten times more than did ethacrynic acid due to the synergistic effect with the benzimidazole ring [116]. 

#### COX-2 enzyme inhibitors

COX-2 is an enzyme responsible for prostaglandin (PG) synthesis. Prostaglandin elevates the invasion and growth of cancer cells and reduces apoptosis [117]. Therefore, COX-2 inhibitors may play an essential role in the prevention and treatment of cancer. Compound **58** was the most active among the pyrimidine-benzimidazole hybrids. The cytotoxic activity of this hybrid was evaluated by an MTT assay, and the results showed moderate activity, with an IC_50_ ranging from 4.30 μM to 9.20 μM against the HT-29, MCF-7, PaCa-2, A549, and PC-3 cancer cell lines compared to that of the standard doxorubicin drug (IC_50_ range = 0.91 μM–5.36 μM). In addition, the compound showed potent COX-1 and COX-2 inhibitory activities with IC_50_ of 2.76 μM and 7.47 μM, respectively, which were comparable to those of indomethacin (IC_50_ = 0.29 μM and 3.82 μM, respectively). On the other hand, it inhibited the phospholipase A2-V enzyme with an IC_50_ of 7.51 μM, while the concentration of the reference drug dexamethasone had an IC_50_ equal to 0.69 μM. Moreover, the docking study indicated that the COX-2 enzyme had good binding affinity (PDB code: 1CX2), with an energy score equal to −11.50 kcal/mol. Moreover, the bromocelecoxib (S-58) reference drug had a binding energy of − 11.93 kcal/mol [118]. 

#### Interaction with bovine serum albumin (BSA)

Bovine serum albumin (BSA) is a macromolecule that targets diseased and cancer cells and is a valuable drug carrier for increasing drug access to target sites, reducing drug side effects, and increasing efficacy. Therefore, substituted triazine-benzimidazole derivatives were synthesized and evaluated as antiproliferative agents. Among the four compounds, **59a-d**, in which the triazine ring was substituted with phenyl, 4-chlorophenyl, 4-fluorophenyl, or piperidine, showed promising antiproliferative activity against the nine tested subpanels of cancer cell lines. The GI_50_ of MIG-MID ranged from 0.72 μM to 2.68 μM. The most potent compound was **59d**, which contains the *para*-chlorophenyl ring on the triazine ring. It exhibited a MIG-MID GI_50_ value of 720 nM. Fluorescence and UV‒visible spectroscopic methods were used to explore the interaction between these triazine-benzimidazole analogs **59a-d** and BSA under physiological conditions. These results indicated that these benzimidazole derivatives **59a-d** could bind strongly to BSA and bind to the target site. Moreover, the distance between the benzimidazole analogs and BSA was in the range of 4–8 nm. This interaction enhances the therapeutic efficacy of drugs for cancer cells and enhances anticancer activity [119]. 

#### Theoretical mechanistic study

A theoretical mechanistic study was carried out for the compounds that showed potent anticancer activity by molecular docking analysis. Molecular docking analysis helps by creating a docking algorithm that incorporates quantum mechanical/molecular mechanical (QM/MM) computations. The extent to which electric charge precision plays a role in protein–ligand docking has been examined [120].

**Fig. 14:**
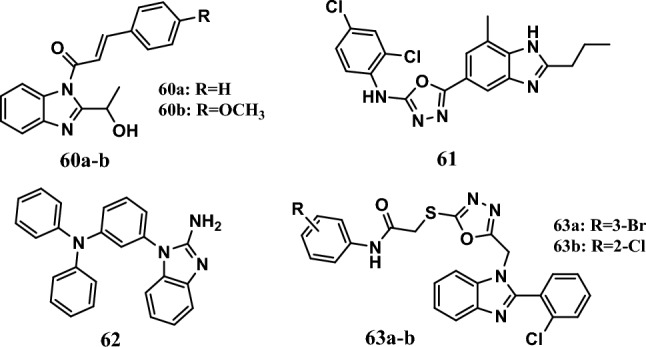
Structures of benzimidazole hybrids **60-63**

Many compounds have been shown to predict EGFR inhibitory activity, as molecular docking studies have shown. For example, a group of 3-(substituted phenyl)-1-[2-(1-hydroxy-ethyl)]-1*H*-benzimidazole-1-yl) prop-2-en-1-one derivatives was designed by molecular docking analysis as EGFR antagonists. *In vitro* anticancer activity was evaluated by a propidium iodide (PI) fluorescence assay in the HCT-116 and H460 cells. Compounds **60a-b** (Fig. 14) were the most potent compounds in this series. The IC_50_ values of compounds **60a-b** ranged from 6.83 µM to 18.16 µM. According to the molecular docking analyses, compounds **60a-b** had docking scores equal to −8.4 kcal/mol and −-8.2 kcal/mol, respectively. The two compounds showed potent affinity for receptor tyrosine kinase. SAR studies have shown the importance of *p*-methoxy phenyl substituents and unsubstituted phenyl rings in chalcones for anticancer activity [121].

Furthermore, a series of benzimidazole 2-amino 1,3,4-oxadiazole hybrids were synthesized and evaluated for their cytotoxic activity by an MTT colorimetric assay. Compound **61** (Fig. 14) was the most cytotoxic compound in this series, with IC_50_ values equal to 4.68 µM, 4.16 µM, and 5.40 µM against the HeLa, MCF-7, and A549 cell lines, respectively, which were comparable to those of doxorubicin, which has IC_50_ values of 2.04 µM, 1.73 µM, and 2.34 µM, respectively. The compound had a safe profile against the normal human embryonic kidney cell line HEK-293, with an IC_50_ equal to 37.25 µM, compared to doxorubicin (IC_50_ = 6.40 µM). A molecular docking study revealed the high affinity of compound **61** for the EGFR kinase domain (PDB_ID: 4H*J*O), for which the docking score was equal to -8.6 kcal/mol, compared to doxorubicin, which has a docking score equal to −8.9 kcal/mol [122].

In addition, compound **62** (Fig. 14) was an example of a benzimidazole/triphenylamine hybrid that showed remarkable cytotoxic activity with an IC_50_ ranging from 14.48 μM to 26.05 μM against the A549, SPC-A-1, MCF-7, HepG2, and H446 cell lines; these results are comparable to those of standard cisplatin, which has an IC_50_ ranging from 21.13 μM to 98.73 μM. In addition, compound **62** showed a good safety profile against normal cell lines, with an IC_50_ >100 μM. Compound **62** exhibited potent interactions with receptor tyrosine kinases (RTKs) with good binding free energies equal to −61.44 kcal/mol, −58.82 kcal/mol, and −51.02 kcal/mol against PDGFRα_T674I, EGFR WT, and EGFR_T790M/L858R, respectively [123].

Among a series of benzimidazole-1,3,4-oxadiazole derivatives, compounds **63a-b** (Fig. 14) had promising anticancer effects on MCF-7 cells, with IC_50_ values ranging from 1.53 μM to 2.08 μM, compared to doxorubicin, which has an IC_50_ equal to 1.45 μM. A docking study was performed on the EGFR protein (PDB 1M17). Compounds **63a-b** had binding energies equal to −8.42 kcal/mol and −8.45 kcal/mol, respectively. Moreover, erlotinib has a binding energy equal to −7.90 kcal/mol [124].

**Fig. 15:**
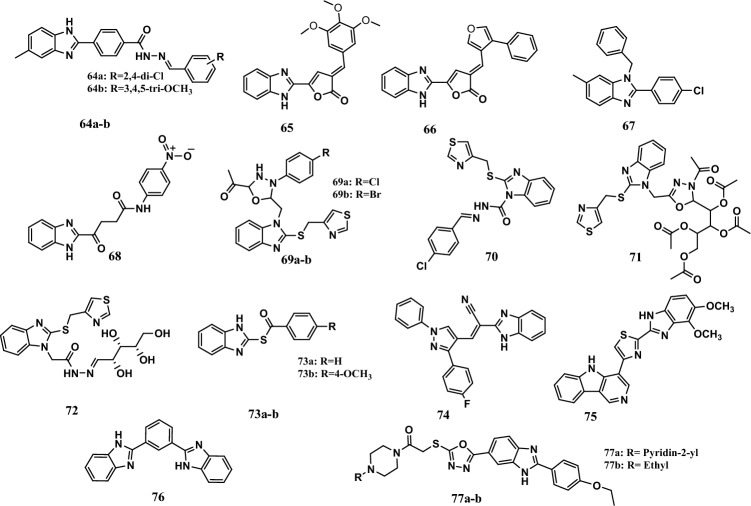
Structures of benzimidazole hybrids **64-77**

In addition, compounds **64a-b** (Fig. 15) were the most active compounds among the series of benzimidazole-hydrazone hybrids. Compounds **64a-b** showed promising growth inhibition effects on sixty different cell lines. Compounds **64a-b** inhibited growth in 50% to 84% of the cells. Molecular docking revealed the high affinity of these compounds for the VEGFR-2 binding site, with binding energies equal to −14.67 kcal/mol and −20.75 Kcal/mol, respectively, while sorafenib had a binding energy equal to −19.10 kcal/mol. In addition, the electron-donating group on the phenyl ring had a greater affinity for VEGFR-2 than the electron-withdrawing group [125].

In addition, two benzimidazole/furanone hybrids, **65** and **66** (Fig. 15), showed moderate anticancer activity according to the MTT colorimetric assay. The hybrids **65** and **66** had IC_50_ values ranging from 9.1 μM to 12.7 μM against the A549, MCF-7, and DU-145 cell lines. Doxorubicin, a standard drug, had IC_50_ values ranging from 0.8 μM to 0.9 μM. Molecular docking studies within the active pocket of the vascular endothelial growth factor receptor (VEGFR; PDB ID: 2QU6) indicated that compounds **65** and **66** had higher affinities for VEGFR, with binding energies equal to −53.32 kcal/mol and −60.21 kcal/mol, respectively, like doxorubicin (binding energy = −53.50 kcal/mol) [126].

Moreover, among synthesized *N*,2,6-trisubstituted 1*H*-benzimidazole derivatives, compound **67** (Fig. 15) was the most potent antiproliferative compound against MDA-MB-231, HepG2, RMS, MCF-7, and C26 with IC_50_ of 2.39 µM, 3.22 µM, 4.83 µM, 5.66 µM, and 3.90 µM, respectively, as compared to paclitaxel (IC_50_ = 1.38 µM, 4.75 µM, 6.13 µM, 2.35 µM, and 3.32 µM, respectively). A molecular docking study of compound **67** in the active sites of dihydrofolate reductase (DHFR-B), fibroblast growth factor receptor 1 (FGFR-1), VEGFR-2, and histone deacetylase 6 (HDAC6) revealed that compounds **67** had good affinities comparable to paclitaxel for all tested proteins targets, with binding energies equal to −10.0 kcal/mol, −9.6 kcal/mol, −8.7 kcal/mol, and −8.6 kcal/mol, respectively, while paclitaxel binding energies were equal to −10.0 kcal/mol, −10.5 kcal/mol, −7.8 kcal/mol, and −8.8 kcal/mol, respectively [127].

In addition, compound **68** (Fig. 15) had remarkable antiproliferative effects on HepG2 and HeLa cells, with growth inhibition percentages equal to 98 % and 92%, respectively, at 50 μM. In addition, the IC_50_ was < 1 μM against the p38α cell line. A molecular docking study showed that compound **68** had significant binding in the active sites of identified p38α MAP kinases [128].

Furthermore, many benzimidazole hybrids with arylidene, 1,3,4-oxadiazoline, or sugar were synthesized from the parent molecule 2-[(4-thiazolyl methyl)thio]-1*H*-benzimidazole. Compounds **69a-b**, **70**, **71**, and **72** (Fig. 15) showed promising anticancer activity against MCF-7 cells, with IC_50_ values ranging from 10.3 μM to 17.9 μM, comparable to that of doxorubicin as a reference drug (IC_50_ = 18.6 μM). The docking study results against CDK2 indicated that compounds **69a-b**, **70**, **71**, and **72** fit well at the active site, with binding energies ranging from −18.61 kJ/mol to −26.41 kJ/mol [129].

Another study was conducted to prove the importance of benzimidazole hybrids as anticancer agents by preparing 2-((imidazole/benzimidazole-2-yl)thio)-1-aryl ethanones. Among them, compounds **73a-b** (Fig. 15) showed promising anticancer activity, with IC_50_ values ranging from 4.53 μM to 12.90 μM against T-47D and MCF-7 breast cancer cell lines according to the sulforhodamine B (SRB) assay. In comparison, staurosporine, a positive control, exhibited IC_50_ values equal to 7.19 μM and 6.67 μM. In addition, compounds **73a-b** bind to the binding site of CDK2 with binding energies equal to − 8.7 kcal/mol and − 8.9 kcal/mol, respectively. Moreover, a CDK2 assay revealed that compounds **73a-b** had moderate inhibitory effects, with IC_50_ values equal to 0.89 μM and 0.69 μM, respectively. In this study, benzimidazole derivatives were shown to have more potent activity than their corresponding imidazole derivatives [130].

In addition, hybrid **74** (Fig. 15) displayed anticancer activity with IC_50_ values equal to 30.9 μM and 32.8 μM against the SW1990 and AsPC1 cell lines, respectively, which were comparable to those of gemcitabine as a positive control (IC_50_ values equal to 35.09 μM and 39.27 μM, respectively). In addition, hybrid **74** showed a safe effect against the noncancerous cell line MRC5, with an IC_50_ equal to 80 μM, which was comparable to that of gemcitabine (IC_50_ = >100 μM). A molecular docking study revealed that hybrid **65** had a high binding affinity for B-cell lymphoma (− 8.65 kcal/mol) [131].

Interestingly, benzimidazole and benzoxazole-linked β-carboline hybrids were synthesized and evaluated for their anticancer activity by MTT assay. All the compounds showed cytotoxic activity, but compound **75** (Fig. 15) showed more potent cytotoxic activity than etoposide, which was used as a positive control. It had IC_50_ values equal to 0.92 µM, 0.72 µM, 0.34 µM, and 1.23 µM against the MCF-7, A549, Colo-205, and A2780 cell lines, respectively. Moreover, etoposide had IC_50_ values equal to 2.11 µM, 3.08 µM, 0.13 µM, and 1.31 µM. According to the docking study, the compounds showed selectivity toward CLK-1 to CLK-4 but not toward EGFR or the ATR kinase. In addition, the thiazole core linked to benzimidazole with a β-carboline nucleus had a significant role in anticancer activity [132].

On the other hand, the hybrid **76** (Fig. 15) of 1,3-bis(benzimidazole-2-yl) benzene showed moderate antiproliferative activity, with IC_50_ values ranging from 13.97 µM to 22.59 µM against the PC-3, HCT-15 and SKLU-1 cell lines; these values were comparable to those of etoposide, which is a standard drug with an IC_50_ ranging from 2.58 µM to 33.87 µM. A molecular docking study showed that compound **76** had a binding affinity equal to − 7.41 kcal/mol. In addition, hydrogen bonds and van der Waals interactions occur with the minor groove of the double-stranded DNA [133].

Furthermore, compounds **77a-b** (Fig. 15) had the most potent antiproliferative activity among the series of benzimidazole-1,3,4-oxadiazole hybrids. Compounds **77a-b** showed more potent activity against HeLa cells than did doxorubicin or hoechst 33342, which were used as positive controls. The IC_50_ values were 0.224 μM and 0.205 μM, respectively. The IC_50_ values of doxorubicin and hoechst 33342 were equal to 14.280 μM and 0.306 μM, respectively. A docking study of the DNA-Topo I enzyme complex showed that the binding energy of compound **77a** was equal to − 7.409 Kcal/mol, which is comparable to that of hoechst 33342 (binding energy= − 4.373 Kcal/mol). In addition, compound **77a** forms a hydrogen bond between the carbonyl group and amino group in the purine ring of the adenine residue (DA113) [33].

Interestingly, a study aimed to prove the anticancer activity of these anthelmintic drugs was performed. The results showed that flubendazole **54d** (Fig. 13) had the same affinity as nocodazole for binding to the CBS, with binding affinities equal to − 7.36 kcal/mol and − 7.25 kcal/mol, comparable to those of the docked nocodazole reference (binding affinities equal to − 7.34 kcal/mol and − 7.04 kcal/mol). Moreover, flubendazole had a high electron density, and one nitrogen of the benzimidazole ring served as an electron-donating site, which formed a hydrogen bond with the colchicine-binding site [134].

Compounds **78a-d** were the most potent derivatives among the 2,5-substituted 1*H*-benzo[d]imidazole hybrids and agreed with Lipinski’s rule. These compounds showed remarkable antiproliferative activity, with IC_50_ values ranging from 5.3 μM to 18.1 μM against HeLa and A549 cell lines, comparable to that of colchicine, which was used as a positive control, with IC_50_ values equal to 17.7 μM and 16.4 μM, respectively. A molecular docking study revealed high binding affinities for both the CBS and the NBS, with binding energy values equal to − 6.72 kcal/mol, − 7.42 kcal/mol, − 7.017 kcal/mol, and − 6.43 kcal/mol, respectively, in CBS. In addition, compounds **78a-d** exhibited high affinities for NBS, with binding energy values equal to − 8.155 kcal/mol, − 8.010 kcal/mol, − 9.509 kcal/mol, and − 9.967 kcal/mol, respectively [135].

**Figure:**
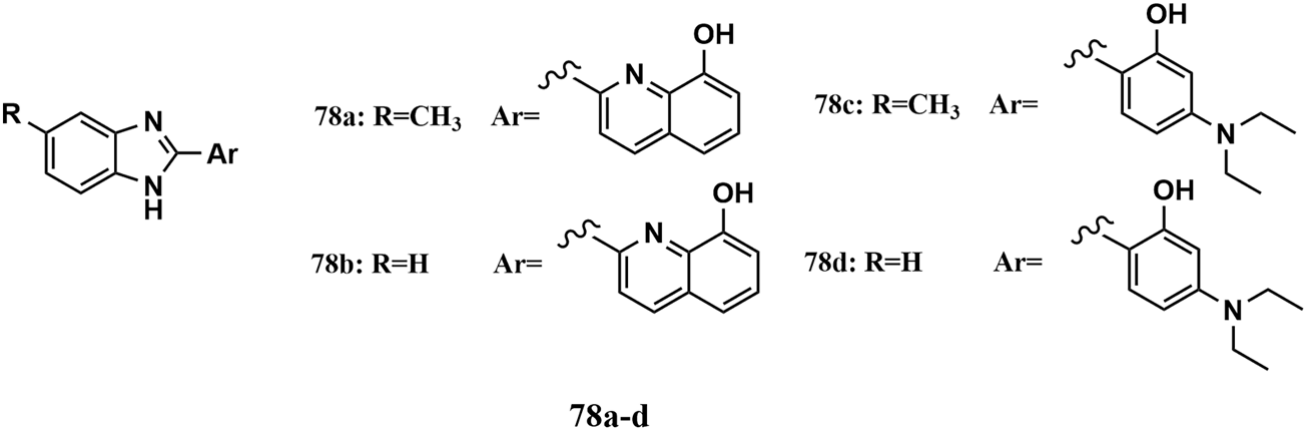

### Mechanism of action of benzimidazole-metal complexes

Benzimidazole complexes with different metal cores were synthesized and evaluated for their anticancer activity from 2016 to 2023. These compounds exert their antiproliferative effects through various mechanisms, as we will see.

#### Induction of apoptosis

**Fig. 16:**
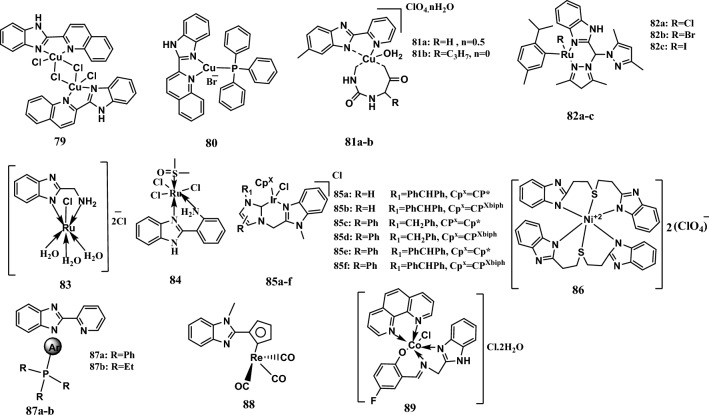
Structures of benzimidazole complexes **79-89**

The 2-(2′-quinolyl) benzimidazole copper complexes **79** and **80** (Fig. 16) showed significant antiproliferative activity, with IC_50_ values ranging from 4.25 μM to 9.24 μM against the SMMC7721, BGC823, HCT1-16, HT-29, and LO-2 cell lines. Cisplatin, a positive control, exhibited IC_50_ values ranging from 6.67 μM to 47 μM. Detection of the interactions of both complexes with calf thymus DNA (CT-DNA) using emission and absorbance spectroscopy methods revealed that complexes **79** and **80** were bound to CT-DNA *via* an intercalative mechanism. In addition, the two complexes exhibited DNA cleavage activity. Moreover, compound **79** induces apoptosis through increasing ROS production and inducing cell cycle arrest at the S phase [136].

Moreover, two complexes, **81a-b** (Fig. 16), which contained a copper(II) core and a 5-methyl-2-(2-pyridyl)benzimidazole ligand, showed promising anticancer activity through the induction of apoptosis in HeLa cells and increasing the level of intracellular ROS that cause damage to CT-DNA, mainly in the presence of ascorbic acid, which was accompanied by the activation of caspase-3 and Bcl-2 family proteins. Complexes **81a-b** exhibited more potent anticancer activity than did the reference drug cisplatin against the A549, PC-3, and HeLa cell lines, with IC_50_ values ranging from 7.88 µM to 16.33 µM. In comparison, cisplatin exhibited an IC_50_ ranging from 15.42 µM to 22.67 µM [137].

In addition, complexes **82a-c** (Fig. 16), with a ruthenium(II) core and a *p*-cymene benzimidazole-based ligand, exhibited potent antiproliferative activity, with IC_50_ values ranging from 6.4 μM to 15.1 μM against MCF-7, MIA, PaCa-2, LNCap, HepG2, and HFF-1 cells. Moreover, cisplatin, a positive control, had an IC_50_ ranging from 5.1 µM to 31.8 μM. The anticancer activity of these compounds was achieved by inducing apoptosis through the intrinsic pathway, cell cycle arrest of MCF-7 cells at the sub-G1 phase, and cell cycle arrest at the G2/M phase. A docking study revealed that complexes **82a-c** could be moderate inhibitors of VEGFR-2, with a gold score ranging from − 34.27 to − 40.54, comparable to that of the standard axitinib drug (gold score = − 58.67). Moreover, western blot analysis of HepG2 cells indicated that the benzimidazole ligand itself and complex **82a** triggered the downregulation of VEGFR-2, Ras, and Akt, but complex **82a** was more potent than the benzimidazole ligand [138].

A water-soluble complex **83** (Fig. 16) formed from a ruthenium(III) core and with a 2-aminomethyl benzimidazole ligand was synthesized and evaluated for its anticancer activity. According to the MTT assay, complex **83** exhibited good activity, with IC_50_ values equal to 18.08 μg/mL and 57.20 μg/mL against the HCT-116 and MCF-7 cell lines, respectively, comparable to the activity of cisplatin as a reference drug (IC_50_ values equal to 18 μg/mL and 22 μg/mL, respectively). It exerted its antiproliferative effect through the induction of apoptosis in MCF-7 and HCT-116 cells, and its DNA damage percentages were 23.1% and 33.1%, respectively. Complex **83** prevented the progression of the cell cycle by decreasing the number of cells that entered the S and G0/G1 phases and increasing the number of cells in the G2/M phase. An *in vivo* study showed that complex **83** prevents the proliferation of female Ehrlich ascites carcinoma (EAC)-bearing mice by decreasing TOPO I levels. Moreover, complex **83** does not cause nephrotoxicity [139].

Interestingly, complex **84** (Fig. 16), which contains a 2-aminophenyl benzimidazole ligand, a ruthenium(III) core, and DMSO, was synthesized and evaluated for its *in vivo* and *in vitro* anticancer activity. Complex **84**_,_ which had IC_50_ values equal to 230 μM and 250 μM, had weak cytotoxic effects on the MCF-7 and Caco2 cell lines, respectively, comparable to cisplatin, which was used as a positive control and had IC_50_ values equal to 22 μM and 18 μM*,* respectively. *In vivo* tests have shown different results than *in vitro*, as complex **84** has potent anticancer effects through the induction of apoptosis accompanied by increased expression of Bax and caspase-3, decreased expression of Bcl-2, inhibition of angiogenesis and prevention of metastasis involving the EAC in a mouse model, and induction of cell cycle arrest at the G2/M phase [140].

Furthermore, the iridium^III^ imidazole and benzimidazole *N*-heterocyclic carbene (NHC) complex **85a-f** (Fig. 16) showed promising anticancer activity, with IC_50_ values ranging from 5.9 μM to 18.2 μM against A549 cells. These compounds were more potent than the reference drug cisplatin (IC_50_ = 21.3 μM). In addition, complexes **85a-f** could bind to BSA and catalyze the oxidation of nicotinamide adenine dinucleotide (NADH) to NAD^+^. Furthermore, flow cytometry analysis of A549 cells treated with the most potent complex, **85f**, revealed that the anticancer activity of complex **85f** was attributed to the induction of apoptosis, cell cycle arrest at the sub-G_1_ and G_2_/M phases, increased ROS production, and altered potential of the mitochondrial membrane [141].

In addition, complex **86** (Fig. 16), formed from the nickel(II) core with the 2-[2-[2-(1*H*-benzimidazole-2-yl) ethylsulfanyl] ethyl]-1*H*-benzimidazole ligand, was synthesized and evaluated for its anticancer activity. Complex **86** showed significant anticancer activity, with IC_50_ values ranging from 5.2 μM to 12.7 μM against MDA/MB-231 cells, in a different time-dependent manner comparable to that of cisplatin, which was used as a positive control (IC_50_ ranging from 10 μM to 25 μM). Moreover, the ligand showed no cytotoxic activity. In addition, complex **86** was found to be safe for the normal cell line Hs27, with an IC_50_ ranging from 38.6 μM to 51.5 μM. The anticancer activity of the complex was induced by apoptosis, hydrolytic DNA cleavage, and fragmentation [142].

Furthermore, two complexes, **87a-b** (Fig. 16), formed from a benzimidazole ligand with a gold(I) core containing either triphenylphosphine or triethylphosphine were synthesized, and their anticancer activity was evaluated *via* an MTT assay. Complexes **87a-b** showed promising antiproliferative activity, with IC_50_ values equal to 1.6 μM and 2.7 μM, respectively, against neuroblastoma SH-SY5Y cells. In addition, they exert their anticancer effects by inducing apoptosis through a caspase-dependent mechanism through the activation of caspase-3 and caspase-9 and through a caspase-independent mechanism through translocation to the nucleus and the elevation of ROS levels [143].

Moreover, complex **88** (Fig. 16), with a rhenium (Re) core and 2-cyclopentadienyl-1-methylbenzimidazole ligand, was the most active complex among the series of Re core complexes. The complex showed potent activity against U251MG cells, with an IC_50_ value of 0.98 µM. In addition, it had good antiproliferative activity against the T98G, SK-N-SH, and Neuro-2a cell lines, with IC_50_ values ranging from 3.99 µM to 19.31 µM. Its cytotoxic activity is due to the induction of apoptosis, increased ROS levels, and cell cycle arrest at the G2/M phase [144].

Recently, complex **89** (Fig. 16), with a cobalt(III) core and benzimidazole-derived imine ligand, demonstrated potent antiproliferative activity compared to its parent ligand against the EAC cells with an IC_50_ of 10 µM. In addition, complex **89** reduced the tumor and showed remarkable tumor-inhibiting activity after five days of intraperitoneal administration in a dose-dependent manner. Furthermore, complex **89** speeds up apoptosis by reducing the vascular supply, which appeared in the anti-angiogenesis and chorioallantoic membrane (CAM) assays [145].

#### DNA damage

Carcinogenesis and its progression are hampered by DNA lesions and repair mechanisms that protect genomic DNA integrity. Most cancer medicines are genotoxic chemicals that cause DNA damage and cause cancer cells to die through apoptosis [146].

**Fig. 17:**
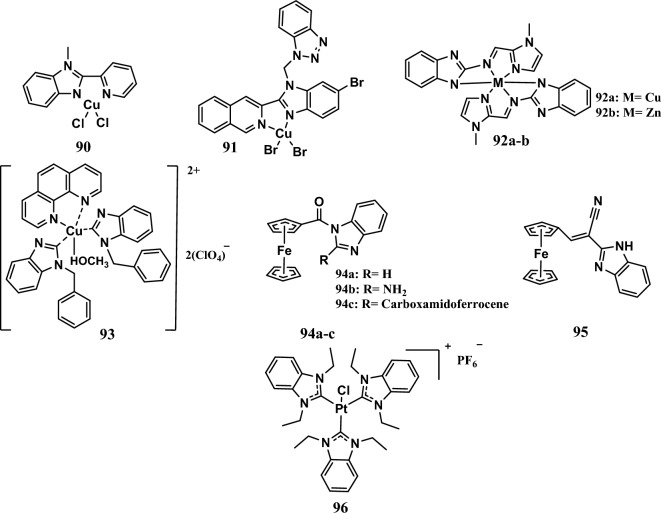
Structures of benzimidazole complexes **90-96**

For example, a series of copper(II) complexes with chelating nitrogen donors of 2-(2-pyridinyl) benzimidazole ligands were developed. Among them, complex **90** (Fig. 17) displayed remarkable cytotoxic activity against A549 cells in this series. It exhibited an IC_50_ of 5.5 μM, while cisplatin, as a reference drug, had an IC_50_ of 3.5 μM. Complex **90** had a synergistic effect, as it was more active than the benzimidazole ligand or free Cu(II). The cytotoxic activity of complex **90** is due to oxidative DNA damage and cell death caused by elevated ROS levels [147].

Furthermore, complex **91** (Fig. 17), which contains a Cu core, exhibited anticancer activity by intercalating CT-DNA, increasing the amount of ROS that damages DNA, and Ca2+ imbalance, which led to homeostasis, decreased ATP production, and induced apoptosis. The complex had IC_50_ values ranging from 10.1 µM to 20.2 µM against the SMMC7721, HCT-116, and TFK-1 cell lines. Cisplatin, a positive control, had IC_50_ values ranging from 3.43 µM to 14.9 µM. Complex **91** showed a greater safety profile against L-02 normal cells, with an IC_50_ equal to 20.1 µM, than did cisplatin (IC_50_ =11.9 µM) [148].

In addition, two complexes, **92a-b** (Fig. 17), formed from a benzimidazole ligand with a Cu or Zn core. The two complexes showed promising to moderate anticancer activity, with IC_50_ values ranging from 3.5 μM to 26.7 μM against the HepG2, SK-MEL-1, HT018, HeLa, and MDA-MB-231 cell lines. The activity of these compounds was comparable to that of cisplatin, which was used as a reference drug (IC_50_ ranging from 3.1 μM to 6 μM). Complex **92a** had a significant anti-metastatic effect by inhibiting the migration and adhesion of HepG2, SK-MEL-1, HT018, HeLa, and MDA-MB-231 cells. Moreover, complex **92a** exhibited significant cleavage of DNA strands, while complex **92b** exhibited moderate cleavage. Interestingly, the *in vivo* chronic toxicity of these compounds against male and female Swiss albino mice indicated that complexes **92a-b** had a safe effect on normal cells [149].

In addition, the Cu complex **93** (Fig. 17) had potent anticancer effects on HCC1428 cells and HUVECs in a dose- and time-dependent manner at concentrations ranging from 2.5 µM to 40 µM. Its IC_50_ ranged from 8.64 µM to 13.50 µM at 24 and 48 h, comparable to that of the ligand, which had IC_50_ values ranging from 29.16 µM to > 400 µM. Moreover, Cu complex **93** significantly decreased cell viability as the concentration increased. The quantity of complex absorbed in the cell membrane increases with increasing concentration, increasing penetration and DNA binding, producing lethal effects in HCC1428 and HUVEC cells. A docking study revealed high binding affinity for the Human serum albumin binding site (PDB:1HZ9), with a binding score equal to −8.44 kcal/mol [150].

Interestingly, ferrocene complexes were synthesized and evaluated for their anticancer activity against HeLa cells. Complexes **94a-c** (Fig. 17) with a benzimidazole moiety showed more potent activity than complexes without a benzimidazole moiety. Complexes **94a-c** had IC_50_ values ranging from 5.10 μM to 11.15 μM, comparable to cisplatin as a standard drug, which showed an IC_50_ equal to 12.08 μM. Interestingly, the increasing number of ferrocene nuclei in complex increased the anticancer activity. Therefore, compound **94c** was the most potent complex with an IC_50_ of 5.10 μM. The principal mechanism of action of complexes **94a-c** is oxidative damage to DNA through increased release of ROS [151].

Moreover, benzimidazole ferrocene complex **95** (Fig. 17) was synthesized and evaluated for its activity by an SRB assay. Complex **95** showed promising activity against A549 cells, with an IC_50_ equal to 16.5 μM. It exerts its antiproliferative effect by interacting with DNA. In addition, the docking study showed that complex **95** had good affinity, with a docking score equal to − 142.757 at the EGFR binding site. Its docking score was higher than that of the standard drug erlotinib (− 132.577) [152].

Recently, among a series of platinum(II) and gold(I) complexes of the benzimidazol-2-ylidene type, a Pt(II) complex **96** (Fig. 17) was the most potent antiproliferative complex on both A2780 wild-type and A2780 cisplatin-resistant ovarian carcinoma cell lines with IC_50_ values equal to 0.63 μM and 1.14 μM, respectively. While cisplatin had IC_50_ values equal to of 0.87 µM and 10.56 µM, respectively. In addition, complex **96** exhibited high stability, with > 90% of the complex remaining unchanged after 72 h of incubation in a cell culture medium. Moreover, results of the cell-free double-stranded (ds) DNA plasmid electrophoretic assay revealed that the complex **96** causes complete destruction of the structure of the dsDNA and only one newly formed band can be seen after 4 h of incubation [153].

#### Glutathione interaction

Complex **97**, in which the organoiridium(III) complex is attached by an azo bond with a 2-(pyridine-2-yl)-1*H*-benzimidazole ligand, exhibited remarkable anticancer activity against the DU-145, A375, HepG2, A549, and MCF-7 cell lines, with IC_50_ values ranging from 10.1 μM to 13.1 μM. Cisplatin, as a positive control, had an IC_50_ ranging from 9.5 μM to 13.3 μM. Complex **97** and ascorbic acid form biomolecules that interact with glutathione, oxidize NADH to NAD^+^, and induce ROS, leading to hemostasis disruption. Ascorbic acid improved anticancer activity by exerting a pro-oxidative effect on the oxidation of GSH to GSSG and increasing ROS levels [154]

**Figure:**
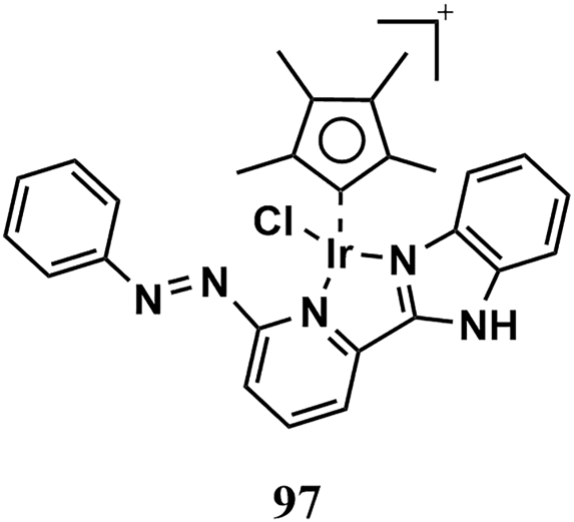

.

#### CO releaser

Carbon monoxide (CO) is a new medication that has received much attention from the scientific community since it can cause mitochondrial malfunction and cancer cell death. Furthermore, as an adjuvant medication, CO can increase the responsiveness of tumor cells to chemotherapy or ameliorate cisplatin-induced nephrotoxicity and doxorubicin-induced cardiotoxicity. However, owing to its higher affinity for hemoglobin and low bioavailability, the direct use of gaseous CO as a medicinal molecule is limited. Several nongaseous CO-releasing molecules (CORMs) with highly varied chemical characteristics have recently been described and utilized to transfer CO to living cells to enhance the efficacy of synergistic therapy and reduce toxicity to normal cells [155].

**Fig. 18:**
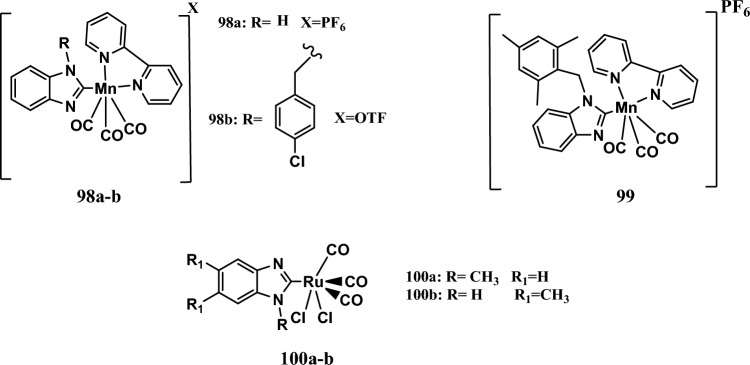
Structures of benzimidazole complexes **98-100**

For example, benzimidazole/CO/manganese complexes **98a-b** (Fig. 18) were synthesized and evaluated as CO releasers. Complexes **98a-b** showed potent anticancer activity against MCF-7 cells, with IC_50_ values equal to 11.4 μM and 9.9 μM, respectively, in comparison to 5-FU as a reference drug (IC_50_ = 13.2 μM) according to an XTT cell proliferation assay [156]. Furthermore, complex **99** (Fig. 18), with trimethyl substituents on the benzyl moiety, was the most active complex among a series of other manganese-based CO-releasing molecules (CORMs). According to the XTT assay method, complex **99** had an IC_50_ equal to 3.22 μM against MCF-7 cells. Moreover, the 5-FU standard exhibited an IC_50_ of 13.25 μM. The anticancer activity increased under UV light, with an IC_50_ equal to 1.79 μM, comparable to that of 5-FU (IC_50_ = 9.45 μM). In addition, myoglobin assays showed that the equivalent of CO release from complex **99** was 1.5 at T_1/2_ 13.9 min [157].

Moreover, compounds **100a-b** (Fig. 18) were the most active complexes among the benzimidazole complexes with the RuII(CO)3}^2+^-core. The compounds showed moderate cytotoxic activity. Compound **100a** had an IC_50_ ranging from 42 to 53 µM, while the concentrations of the KP1019 reference drug had an IC_50_ equal to 79 µM and 44 µM against the SW480 and CH1/PA-1 cell lines, respectively. *In vivo* anticancer studies of a murine colon cancer CT-26 tumor model showed that **100b** significantly reduced tumor growth. Both compounds **100a-b** had moderate release of CO as a CO ligand bound to Ru [158].

#### ER stress pathway

The endoplasmic reticulum (ER) is the prominent organelle in the cell and is responsible for various cellular tasks, such as protein folding and maturation, as well as cellular homeostasis. The unfolded protein response (UPR), which restores homeostasis or activates cell death, is triggered by ER stress for many reasons. ER stress helps restore homeostasis and enhances the tumor survival and growth in the surrounding environment. In addition, apoptosis can result from unresolved ER stress in a caspase-dependent manner [159]. Pyridyl-fluoro benzimidazole-derived copper complex **101** showed more anticancer activity than its ligand, as proven by the MTT results. Complex **101** had IC_50_ values equal to 10.0 µM and 22.4 µM for 72 h against the DU-145 and SPC212 cell lines, respectively, while the IC_50_ ligand was >100 µM [160]. 

#### Inhibition of the membrane water and glycerol channel aquaporin (AQP3)

AQPs are transmembrane proteins that allow glycerol and water to diffuse across cell membranes and are essential for water and energy balance, fluid secretion, and epidermal hydration. The overexpression of aquaporins (AQPs) in cancer and their abnormal expression in many human malignancies, including skin cell carcinomas and melanoma, have led to the use of inhibitors of these proteins as anticancer agents. Metal compounds and small molecules were discovered to be potential therapeutic leads that require *in vivo* confirmation [161].

Complexes **102a-c** had a Au(III) core and pyridyl benzimidazole ligands. The IC_50_ values ranged from 5 μM to 41 μM against the SKOV3, MCF-7, and A375 cell lines, comparable to that of Auphen, which is a standard drug (IC_50_ ranging from 0.80 μM to 7 μM). Complex **102b** selectively inhibited the membrane water and glycerol channel aquaporin (AQP3) in human red blood cells, with an IC_50_ value equal to 0.82 μM, which was comparable to that of Auphen (IC_50_ = 0.80 μM) [162]. 

#### Increasing the level of cations in cells

Interestingly, the silver core **103a-c** complex exhibited potent cytotoxic activity due to an increase in the silver cation concentration in cells, which led to metabolic and respiratory mechanism failure. Complexes **103a-c** exhibited more potent anticancer activity than did their corresponding benzimidazole ligands, with IC_50_ values ranging from 4.22 μM to 10.3 μM against the MDA-MB-231 and HCT-116 cell lines. Moreover, their ligands exhibited decreased activity, with IC_50_ values ranging from 25.51 μM to 34.21 μM, comparable to those of the reference drugs tamoxifen (IC_50_ = 8.20 μM against MDA-MB-231 cells) and 5-FU (IC_50_ = 5.5 μM against HCT-116 cells). All the complexes showed a safe effect against EAhy926 normal cells, with IC_50_ values ranging from 32.20 μM to 112.47 μM, comparable to that of betulinic acid, which was used as a positive control, with an IC_50_ equal to 29.3 μM. An SAR study showed that the activity of these complexes was enhanced by increasing the length of the side chain, which in turn increased the lipophilicity of the benzimidazole ring [163]. 

## Conclusion

Benzimidazole is one of the most important scaffolds that has potent anticancer activity due to its structure similarity to nucleoside. In this review, we have listed the benzimidazole hybrids or metal complexes having antiproliferative activity from 2016 to 2023. The examples discussed in this review explained the different mechanisms of action by which benzimidazoles exerted their antiproliferative effects. We have also highlighted the rationale for incorporating benzimidazole core with other active pharmacophores or in metal complexation to obtain multitargets anticancer agent. In summary, benzimidazoles could act as hydrogen donors or acceptors and bind to different drug targets so benzimidazole is a promising scaffold for the development of new anticancer agents having drug-like properties and act as multitarget anticancer agents for developing drug candidate. In addition, this is the future direction for building novel therapeutic molecules and developing new anticancer benzimidazoles.

## Data Availability

The data and materials used for our figures and tables are available upon request because this is a review.
